# HIV prevention among youth: A randomized controlled trial of voluntary counseling and testing for HIV and male condom distribution in rural Kenya

**DOI:** 10.1371/journal.pone.0219535

**Published:** 2019-07-30

**Authors:** Esther Duflo, Pascaline Dupas, Thomas Ginn, Grace Makana Barasa, Moses Baraza, Victor Pouliquen, Vandana Sharma

**Affiliations:** 1 Massachusetts Institute of Technology, Cambridge, MA, United States of America; 2 Stanford University, Stanford, CA, United States of America; 3 Innovations for Poverty Action, Busia, Kenya; 4 Evidence Action, Nairobi, Kenya; 5 Paris School of Economics, Paris, France; 6 Harvard T.H. Chan School of Public Health, Boston, MA, United States of America; International AIDS Vaccine Initiative, UNITED STATES

## Abstract

**Objective:**

Voluntary Counseling and Testing for HIV (VCT) and increasing access to male condoms are common strategies to respond to the HIV/AIDS pandemic. Using biological and behavioral outcomes, we compared programs to increase access to VCT, male condoms or both among youth in Western Kenya with the standard available HIV prevention services within this setting.

**Design:**

A four arm, unblinded randomized controlled trial.

**Methods:**

The sample includes 10,245 youth aged 17 to 24 randomly assigned to receive community-based VCT, 150 male condoms, both VCT and condoms, or neither program. All had access to standard HIV services available within their communities. Surveys and blood samples for HSV-2 testing were collected at baseline (2009–2010) and at follow up (2011–2013). VCT was offered to all participants at follow up. HSV-2 prevalence, the primary outcome, was assessed using weighted logistic regressions in an intention-to-treat analysis.

**Results:**

For the 7,565 respondents surveyed at follow up, (effective tracking rate = 91%), the weighted HSV-2 prevalence was similar across groups (control group = 10.8%, condoms only group = 9.1%, VCT only group = 10.2%, VCT and condoms group = 11.5%). None of the interventions significantly reduced HSV-2 prevalence; the adjusted odds ratios were 0.87 (95% CI: 0.61–1.25) for condoms only, 0.94 (95% CI: 0.64–1.38) for VCT only, and 1.12 (95% CI: 0.79–1.58) for both interventions. The VCT intervention significantly increased HIV testing (adj OR: 3.54, 95% CI: 2.32–5.41 for VCT only, and adj OR: 5.52, 95% CI: 3.90–7.81 for condoms and VCT group). There were no statistically significant effects on risk of HIV, or on other behavioral or knowledge outcomes including self-reported pregnancy rates.

**Conclusion:**

This study suggests that systematic community-based VCT campaigns (in addition to VCT availability at local health clinics) and condom distribution are unlikely on their own to significantly reduce the prevalence of HSV-2 among youth.

## Introduction

The HIV/AIDS pandemic remains a significant global health problem. An estimated 36.9 million people globally were living with HIV in 2017, with 70% in sub-Saharan Africa [1]. Despite two decades of prevention efforts, 1.8 million new infections occurred in 2017 [1]. Globally, young people are disproportionately affected by HIV—in 2017, 590,000 new HIV infections occurred among youth aged 15–24, representing over 30% of all new infections [2]. Recent estimates suggest approximately 30% of young women and 30% of young men in sub-Saharan Africa in 2017 had basic information about HIV prevention [3], and in 2013 only 15% of young women and 10% of young men in the region knew their HIV status [4].

Voluntary Counseling and Testing for HIV (VCT) plays an important role in the worldwide response to the HIV/AIDS pandemic, serving as a crucial entry point to HIV/AIDS treatment and care [5]. VCT’s potential contribution as an HIV prevention strategy has also been emphasized. It is hypothesized that through the process of learning one’s HIV sero-status, in addition to receiving individualized risk reduction counseling, VCT could help individuals reduce sexual risk taking behaviors and protect themselves and their partners from HIV and other sexually transmitted infections such as Herpes Simplex Type 2 (HSV-2) [6–9].

However, previous studies assessing the impact of VCT as a prevention intervention have shown inconsistent results [10–16]. For example, several studies have found reductions in risky sexual behaviors due to VCT [10] that are, in some studies, more pronounced in those testing HIV positive [11–14], while others have found no association between VCT and changes in sexual behavior [15, 16]. There have been concerns that VCT could lead to disinhibition or an increase in sexual risk behaviors amongst those testing negative for HIV, and this has been reported in at least one study [17]. Several randomized controlled trials (RCTs) assessing the impact of VCT have been conducted including one in Zimbabwe which found no effect of VCT on HIV incidence [18], and one in Kenya, Tanzania and Trinidad which reported reductions in unprotected intercourse in participants receiving VCT but did not assess biological outcomes [13]. A large RCT of a multi-component HIV prevention program (Project Accept) including community-based VCT in several sites in sub-Saharan Africa and in Thailand found significant increases in HIV testing among men and women, and a reduction in HIV incidence among women older than 24 years, but not among youth [19]. A recent meta-analysis found that people who received VCT were significantly more likely to report reducing their number of sexual partners compared to those who did not receive VCT, and were also more likely to report protected sexual intercourse if they tested positive for HIV [11].

Few of these studies disaggregated findings by age and overall there is limited evidence on the impact of VCT on sexual risk behaviors specifically among youth [20, 21]. Nevertheless there is a body of evidence reporting barriers to HIV testing among youth including lack of HIV/AIDS knowledge, lack of awareness of available services, belief that own risk is low, concerns about confidentiality, fear, and financial burden [22–26]. Youth may also not regularly access health care where traditional VCT services are often based [21]. Alternative modalities of VCT delivery such as home-based [15, 27], mobile [28, 29] and self-testing [30] may provide increased confidentiality and address other barriers faced by youth. For example, a secondary analysis of Project Accept data demonstrated that community-based VCT significantly increased HIV testing among youth, especially young men with high-risk behavior [31].

Youth additionally face barriers in accessing and using condoms, another important strategy in the worldwide response to HIV/AIDS [32]. A systematic review of 62 evaluations of condom interventions implemented in Africa and Asia since 1998 reported that few of the included studies used randomized designs or control groups and most measured self-reported condom use as the primary endpoint [33]. Of the fourteen studies that focused on increasing reported condom use among youth, only 8 showed significant, but small increases (6–19% absolute increase) [33]. There remain significant evidence gaps on how to most effectively increase condom use, including whether condoms should be distributed for free or made available for a small fee [34]. Previous studies on other types of preventive goods such as bednets and chlorine have demonstrated that free distribution increases adoption [35–37]. In Kenya, supply side challenges relating to cost and access to condoms remain a barrier that contributes to low condom use, even when condoms are available at multiple sites both freely and for sale in shops within the community [38], and these barriers are even higher among youth [32]. However, to our knowledge, there are no systematic evaluations assessing the impact on biological outcomes of programs to address those supply side barriers including through the free distribution of a large quantity of condoms among youth. It is also unclear whether increased access to condoms might increase the potential impact of VCT, enabling youth to put into practice safer sexual behaviors.

To fill some of the above gaps, this paper presents findings from a randomized controlled trial of interventions to increase access to HIV testing and male condoms in rural Kenya among a population aged 17–24 years. The study assesses the impact of community-based VCT, direct free male condom distribution, and both interventions together (provided in addition to existing VCT and condoms available locally) on biological (HSV-2 and HIV infection) and self-reported behavioral outcomes among youth. The two interventions aimed to remove logistic and other barriers that limit youth access to HIV prevention programming with the goal of reducing risky sexual behavior and potentially decreasing transmission of sexually transmitted infections such as HSV-2 and HIV.

## Methods

### Study design and participants

The study is a four arm, unblinded, individually randomized controlled trial implemented between 2009 and 2012 in four districts of Kenya’s Western Province (Butere, Mumias and Bungoma South and Bungoma East). The districts span an area of approximately 50,000 square kilometers. The HSV-2 prevalence in Western Province Kenya in 2007, just before the start of the trial, was reported to be 37.8% in the 2007 Kenya AIDS Indicator Survey (KAIS) [39]. Women had a significantly higher prevalence of HSV-2 (42%) compared to men (26%) [40]. In addition, between the ages of 15 and 24 years, HSV-2 prevalence increased rapidly from 7% to 34% in women and 3% to 14% in men [40]. The prevalence of HIV in Western Province was reported to be 5.4% in the 2007 KAIS [39], and dropped to 4.7% in the 2012 KAIS [41].

The study sample is composed of youth who were 17 to 24 years old in 2009 at the start of this study. These sampled youth were part of an earlier study conducted by Duflo, Dupas and Kremer [42] in 328 primary schools in the same study site. The sample for the earlier study, a clustered randomized controlled trial assessing a teacher training program in the government’s HIV/AIDS curriculum and the distribution of free uniforms, included 19,289 students enrolled in Grade 6 in one of the 328 schools in 2003. To assess the impact of the teacher training and uniforms programs, the study team attempted to track all individuals at their initial locations between March 2009 and July 2010. Approximately 55% of them could be tracked during this first round of tracking (called “regular tracking”). The individuals who could be surveyed in that regular tracking (10,245 individuals, 46% of them girls) form the sample for the present study. Eligibility criteria for participation in this current trial include: 1) participation in the previous RCT, 2) residency in the study districts at baseline, and 3) ability to provide informed consent. Given the inclusion criteria of the previous trial, the current study sample therefore includes youth who attended at least Grade 6. The study population was representative of 85% of all youth aged 17–24 in the Western Region of Kenya in 2009 according to Demographic and Health Survey data [43].

The trial was implemented by the Abdul Latif Jameel Poverty Action Lab (J-PAL) at the Massachusetts Institute of Technology (MIT), in partnership with Innovations for Poverty Action (IPA) Kenya. Individuals enrolled in the trial were randomly assigned to one of four study arms–a control arm and three treatment arms (community-based VCT, free supply of condoms, community-based VCT and free supply of condoms). Data was collected from enrolled individuals at baseline and approximately 24 months after baseline. The factorial study design allows assessment of the impact of each intervention alone as well as the combination of the two programs together. Given the complementarity of the two interventions (access to knowledge about HIV transmission and one’s own HIV status versus access to condoms which enable youth to decrease unprotected intercourse), the impact of the combine interventions may be greater than the individual programs.

### Randomization and masking

Random assignment of individuals (25% to each arm) was conducted using a sequence of random numbers generated in Stata using a reproducible seed. It included stratification by gender, primary school, and information collected from study participants earlier: whether they were enrolled in secondary school as of July 2007, and, for females, whether they had ever been pregnant as of July 2007. Stratifying by primary school ensured that groups were balanced with respect to the different treatment arms in the Duflo, Dupas and Kremer study [42]. The randomization process was carried out by the principal investigators prior to the start of the baseline using the list of all participants who were enrolled in the 2003 study (19,289). Given this randomization procedure, the field team did not implement a random allocation sequence in the field. Blinding of sampled individuals and baseline enumerators in treatment arms was not possible, as sampled individuals were informed of their treatment assignment and invited to participate in the intervention immediately after the baseline data collection. At follow up enumerators were blinded, though there is the possibility that they may have observed materials linked to intervention assignment. Laboratory staff members involved with testing of blood samples were blinded to intervention assignment.

### Procedures

Data were collected from the sample at baseline from March 2009 to July 2010. The interventions were provided immediately after the completion of the baseline questionnaire. The follow up data collection was conducted between April 2011 to May 2013. Note that the baseline of this trial (administered between March 2009 and July 2010) constitutes the follow up survey for the Duflo, Dupas and Kremer study [42].

#### Interventions

VCT was conducted in accordance with the Kenya National HIV Testing Guidelines [44] and was offered onsite within respondents’ communities or within their homes immediately after the baseline survey to all respondents randomized to one of the VCT intervention groups. All field staff providing VCT were certified VCT counselors and trained by the National AIDS and STI Control Program (NASCOP). For those who provided informed consent, all three components (pretest counseling, HIV testing and post-test counseling) were performed in a single sitting, as is done at government VCT sites in Kenya. The HIV testing procedure involved serial testing with the rapid HIV tests Determine (Abbott Diagnostics), Bioline (Standard Diagnostics, Inc) and Uni-Gold (Trinity Biotech) as per the National Guidelines. The main difference between the VCT intervention provided in this trial and standard VCT available at local health facilities involved the provision of the services within communities near or at respondent’s homes, thus removing some of the barriers to testing such as travel time and cost, as well as social costs of visiting a publically known HIV testing center. Respondents who tested positive for HIV were referred to the closest clinic with the capacity to provide HIV/AIDS care and treatment. Dried blood spots were collected from a random sample of 10% of the respondents and were tested at the National HIV Reference Lab (NHRL) at Kenyatta National Hospital in Nairobi for quality control. All respondents, regardless of treatment group were offered VCT during the follow up survey. The study protocol for HIV testing during the VCT sessions was modified during the follow up data collection period to meet revised Kenya National HIV testing guidelines. The revised protocol involved serial testing with Determine and Bioline only. Discordant samples were confirmed by ELISA testing at the NHRL.

Respondents randomly selected to one of the condom intervention arms (free condoms or VCT plus free condoms) were offered 50 packages containing 3 condoms each (Trust brand) free of charge at the end of the baseline survey. Respondents could take all or a share of the condoms. If they took all and did not give them away, these 150 condoms would be sufficient to cover sexual activity for a full year for individuals who have sexual intercourse every other day, and for three years for those who have sexual intercourse once a week.

Respondents assigned to the VCT plus free condoms arm received the VCT session nearby or in their homes as well as the 50 packages of Trust condoms. Respondents in the control arm were not offered community-based VCT or condoms. Respondents in all study arms were able to access VCT and condoms freely at local health facilities and purchase condoms at local shops. At follow up, study participants in all four trial arms were offered community-based VCT, regardless of whether they had previously been tested.

#### Data collection

Data at baseline and at 2-year follow up were collected using paper-based surveys administered in Swahili by trained male and female enumerators from the study areas. The baseline questionnaire consisted of several modules: 1) a Knowledge, Attitudes and Skills module that included questions regarding HIV prevention and attitudes towards the disease; 2) a behavioral module that included questions on sexual behavior, past and current sexual partners, marriage and childbearing and 3) a module comprising questions relating to socio-economic variables such as education and income, as well as general attitudes and perceptions. In addition, detailed contact information was recorded in order to facilitate follow-up and reduce potential attrition between baseline and follow up. Blood samples were also collected for HSV-2 testing.

For the follow up data collection, the study team attempted to survey all study participants starting in April 2011, roughly two years post-intervention, as well as collect blood samples for HSV-2 testing. The field team first invited all respondents to attend “camps” organized at local health facilities throughout each district. The camps were held for specific periods of time (1–2 weeks) at each venue and respondents were invited through letters delivered in person or to their relatives and neighbors. Field officers then attempted to track respondents who had not visited the camp. Those were divided into two types: T1 for those with at least some location and contact information obtained from a relative or teacher and T2 for which we were not able to obtain any location or contact information. 25% of the Type 1 respondents and 10% of Type 2 respondents were randomly selected for further, intensive tracking efforts. The intensive tracking phase took place between January 2013 and May 2013 and consisted of both local and long-range visits. Teams of field officers and lab technicians were sent to various locations (including those outside of our initial study area, such as Nairobi and Mombasa) in order to individually track, administer the follow-up survey, and conduct blood draws with the target respondents at their current location. Respondents were reimbursed for transport and given a gift (a segment of fabric) for completing the survey.

### Outcomes

The pre-specified primary outcome measure was HSV-2 prevalence, measured through blood tests at baseline and at follow up. HSV-2 infection was chosen as the primary outcome given its relatively high prevalence in Kenya [40] compared to other STIs [45], and the fact that HSV-2 is rarely transmitted through means other than sexual intercourse, making it a good biomarker of risky sexual intercourse. Secondary outcomes included HSV-2 incidence, HIV prevalence, sexual risk behaviors, HIV testing rates, HIV knowledge and attitudes, pregnancy rates and number of children.

All blood samples were tested at the AMPATH (Academic Model Providing Access to Healthcare) Reference Laboratory at Moi University School of Medicine in Eldoret, Kenya. HSV-2 prevalence was assessed by analysis of serum samples collected during baseline and follow up, using the KALON HSV Type 2 IgG ELISA test (KALON Biologicals, Guildford, UK). Positive samples were confirmed and indeterminate samples were retested. Persistently indeterminate specimens (n = 44) were classified as negative as per laboratory guidelines. Note that excluding the indeterminate samples from the analysis has no effect on the findings. As part of quality control procedures, a random 10% sample of specimens was also tested for HSV-2 at the NHRL in Nairobi, Kenya. Respondents testing positive for HSV-2 were referred to the nearest clinic with capacity to treat sexually transmitted infections (STIs).

HSV-2 incidence was calculated including only respondents who were negative at baseline and dividing new HSV-2 cases in each treatment arm by the total person-years of exposure, where person-years of exposure is calculated as the total time from baseline test to the follow up negative result if the person remained negative, or as half the time between the baseline test and the positive test.

At follow up, all respondents were offered VCT. HIV prevalence at follow up is therefore assessed using the VCT test results which are described in the intervention section above and involved HIV rapid testing using Determine and Bioline.

Behavioral outcomes were assessed using questionnaires as described in the data collection section. Uptake of HIV testing was calculated as the percentage of respondents who reported having VCT at least once in their lifetime, more than once in their lifetime, and more than twice in their lifetime. Access to condoms was assessed as the proportion of respondents who reported ever receiving condoms for free during their lifetime. A number of sexual behaviors were also assessed. This included the proportion of respondents who reported ever having had sex, the proportion who reported not using a condom at last sexual intercourse (among those who have had sex), and the proportion who ever had unprotected sex with a non-monogamous partner. The reported lifetime number of sexual partners and the number in the last 6 months was also assessed, The proportion of respondents who reported having a sexually transmitted infection or self-sores, ulcers or abnormal discharge in the last 12 months was determined. HIV knowledge outcomes were measured in two ways: 1) the proportion of respondents who correctly named at least three ways to prevent HIV, and 2) the proportion who correctly answered three HIV knowledge questions (1- Can HIV be transmitted to a baby in the womb?; 2- Can HIV be transmitted to a baby during breastfeeding?; 3- Can HIV spread through mosquito bites?). The proportion of respondents with positive attitudes towards people living with HIV (defined as: Agreed that people with HIV/AIDS should be treated the same as people without HIV/AIDS, disagreed that prostitutes or promiscuous men are responsible for spreading HIV, and disagreed that HIV was punishment for bad behavior) was also measured. Finally, the proportion of respondents who have ever been pregnant, or have ever made their partner pregnant was calculated, and the number of children was also assessed.

### Statistical analysis

Sample size calculations for HSV-2 prevalence were conducted assuming a type 1 error (alpha) of 0.10 and power (1-beta) of 0.8, for the sample of 10,245 youth who were found at follow up of the 2003 study and assuming that 6.5% would be lost to follow up over the study period. Using the measured prevalence of HSV-2 at baseline (7.3% overall, 9% among girls, 6% among boys) the study was powered to detect a 19% reduction in HSV-2 prevalence for the full sample, a 26% reduction for females, and 32% reduction for males when evaluating the impact of one HIV prevention program only (VCT or condom distribution) vs. control. The minimum detectable effect size (% difference in HSV-2 prevalence) of the combined interventions (VCT and condoms) versus one intervention only was higher at 21% for the full sample, 36% for females and 45% for males. The *ex post* minimum detectable effect size is higher however, because the standard errors are increased by the weighting for intensive tracking.

We compared women’s and men’s characteristics at baseline using descriptive statistics. Weighted follow up rates (effective final tracking rates), taking into account the two tracking phases, were calculated to provide an estimate of the share of the total sample for which the data are representative. To estimate the effect of the interventions on outcomes measured at 24 month follow up, an intention-to-treat analysis was conducted. This included constructing weighted logistic regression models to compare outcomes in each treatment arm to the control. Analysis weights are used to account for the survey tracking strategy, and are calculated as Total Eligible / Total Surveyed within treatment group and within gender when applicable. Results are reported for all respondents who were surveyed and consented to a blood draw, and were examined separately for men and women. In the primary analysis, we include HSV-2 status or secondary outcomes at follow up as the dependent variable and control for age categories, months between baseline and follow up, the 2003 treatment arm, and variables used for stratification: gender, primary school, whether they were enrolled in school in 2007, and whether females were pregnant in 2007. Adjusted odds ratios and their 95% confidence intervals are presented for each outcome comparing the prevalence in each intervention arm versus the control arm as per the pre-planned ITT analysis

We also conducted secondary analyses where we tested for heterogeneity in the results by gender, whether the respondent had children at baseline, and the respondent’s baseline belief about the likelihood of current HIV infection. We also assess results by 2003 treatment arm as a robustness check.

### Ethical considerations

Written informed consent was obtained from each participant and separately for the survey, the HSV-2 testing, and the anonymous HIV testing. Assent was obtained from participants who were less than 18 years of age along with parental consent to participate in the trial. The trial protocol received ethical clearance from the Massachusetts Institute of Technology (MIT), Stanford University, and the Kenya Medical Research Institute (KEMRI). The trial was registered at socialscienceregistry.org (https://www.socialscienceregistry.org/trials/170). In addition, the trial was registered retrospectively at clinicaltrials.gov (NCT03868644) after study completion, once the authors became aware of this requirement for publication. The authors confirm that all ongoing and related trials for this intervention are registered; there are no ongoing trials related to this study.

## Results

**Fig 1:**
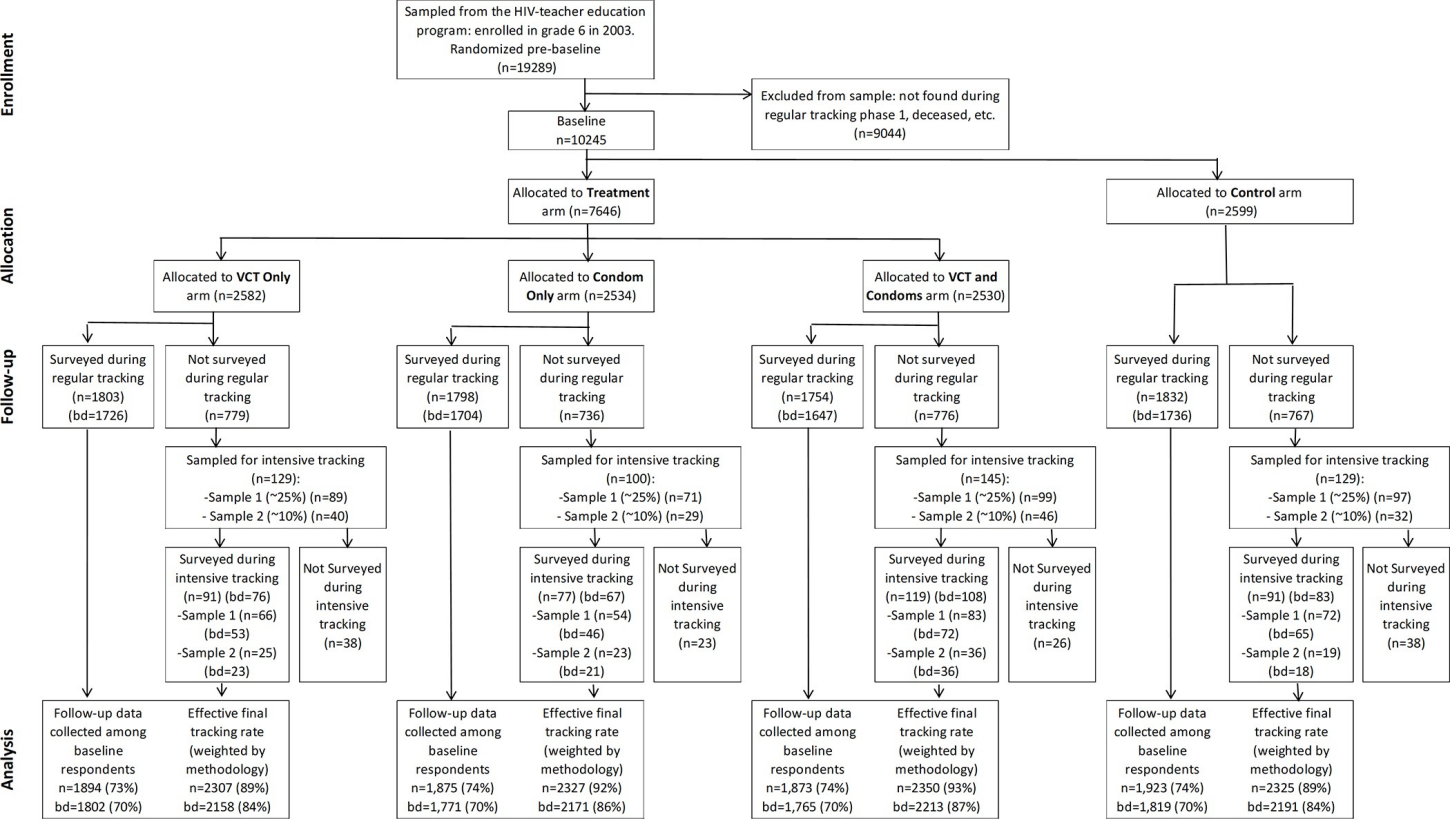
Participant flow diagram. Note: "bd" gives the number of respondents for whom a blood draw was successfully taken. a: the effective final tracking rate combines regular and intensive tracking rates, and provides an estimate of the share of the total sample for which the data are representative.

In total, 10,245 youth who were 17 to 24 years old in 2009 at the start of this study were eligible for participation. Of these 10,245 participants, 2,582 had been randomly assigned to the VCT only arm, 2,534 randomly assigned to the condoms only arm, 2,530 randomly assigned to the VCT and condoms arm and 2,530 randomly assigned to the control arm (Fig 1). During the follow up survey, 1,803 respondents (70%) in the VCT only arm, 1,798 respondents (71%) in the condom only arm, 1,798 respondents (71%) in the VCT plus condoms arm and 1,832 respondents (70%) in the control arm were surveyed during regular tracking. Of those selected for intensive tracking among the remainder, 77% of T1 respondents and 70% of T2 respondents were located and surveyed. This brings the overall weighted follow up rate (effective tracking rate) to 91% (89% in the VCT arm, 92% in the condoms arm, 93% in the VCT plus condoms arm, 89% in the control arm). Among those who were not surveyed, 94% could not be found, 4% declined the survey, 1% were deceased, and less than 1% were excluded for other reasons including mental illness or incarceration. 76% of respondents who completed the follow up survey consented to VCT, and 95% consented to a blood draw for HSV-2 testing. The number of participants missing from the follow up did not differ significantly between treatment and control groups.

Baseline characteristics of women and men across the four trial arms who were surveyed during the follow up data collection were balanced at baseline ((See Table 1). Roughly half of the sampled respondents were between 19 and 20 years of age at baseline, approximately 20% were married and around 42% had ever been tested for HIV (this was higher among women than men). Only about one third of the sample could correctly answer all three of the HIV knowledge questions and approximately 13% had accepting attitudes towards people living with HIV/AIDS.

**Table 1:** Baseline characteristics of those surveyed at follow up.

	All				Women				Men			
	Control	Condom only	VCT only	Condom and VCT	Control	Condom only	VCT only	Condom and VCT	Control	Condom only	VCT only	Condom and VCT
Number of individuals	1923	1875	1894	1873	877	823	842	822	1046	1052	1052	1051
Age at baseline <19	504 (24.2%)	519 (27.2%)	509 (28.9%)	490 (26.0%)	301 (32.6%)	286 (32.7%)	299 (41.2%)	251 (31.8%)	203 (17.1%)	233 (22.8%)	210 (18.5%)	239 (22.2%)
Age at baseline 19–20	968 (52.4%)	927 (52.2%)	932 (46.7%)	939 (50.8%)	450 (52.3%)	428 (56.1%)	412 (43.2%)	447 (55.8%)	518 (52.8%)	499 (49.2%)	520 (49.9%)	492 (47.1%)
Age at baseline 21–22	391 (20.0%)	379 (18.4%)	393 (20.4%)	376 (19.5%)	111 (13.9%)	98 (10.1%)	111 (12.7%)	109 (11.1%)	280 (24.9%)	281 (24.8%)	282 (26.7%)	267 (25.3%)
Age at baseline >22	60 (3.4%)	50 (2.2%)	59 (4.0%)	68 (3.6%)	15 (1.3%)	11 (1.0%)	20 (3.0%)	15 (1.3%)	45 (5.2%)	39 (3.2%)	39 (4.8%)	53 (5.4%)
Not enrolled in school	977 (52.2%)	939 (49.8%)	945 (48.1%)	954 (49.9%)	459 (52.7%)	432 (49.3%)	447 (48.6%)	463 (57.0%)	518 (51.9%)	507 (50.0%)	498 (47.6%)	491 (44.6%)
Total years of schooling <10	496 (25.4%)	462 (23.9%)	506 (25.9%)	512 (26.3%)	281 (33.1%)	236 (28.8%)	269 (28.9%)	257 (31.3%)	215 (19.0%)	226 (20.0%)	237 (23.2%)	255 (22.6%)
Total years of schooling 10–11	480 (24.6%)	484 (23.6%)	462 (23.6%)	412 (21.5%)	206 (21.2%)	208 (22.0%)	207 (23.4%)	202 (22.2%)	274 (27.6%)	276 (24.7%)	255 (23.9%)	210 (20.5%)
Total years of schooling 12–13	833 (45.7%)	825 (46.8%)	810 (43.9%)	825 (46.9%)	364 (44.1%)	351 (45.2%)	334 (44.0%)	335 (44.5%)	469 (46.8%)	474 (48.4%)	476 (43.9%)	490 (49.1%)
Total years of schooling >13	101 (4.3%)	91 (5.7%)	102 (6.6%)	102 (5.3%)	18 (1.5%)	24 (4.0%)	24 (3.7%)	18 (1.9%)	83 (6.6%)	67 (7.0%)	78 (9.0%)	84 (7.8%)
Blood test: HSV-2 positive	103 (5.5%)	94 (4.8%)	100 (6.3%)	104 (5.4%)	64 (7.7%)	60 (7.1%)	60 (8.3%)	61 (6.4%)	39 (3.4%)	34 (2.8%)	40 (4.6%)	43 (4.3%)
Currently married	395 (21.2%)	376 (18.2%)	389 (20.3%)	384 (18.3%)	244 (28.9%)	225 (24.7%)	238 (27.3%)	231 (25.6%)	151 (14.5%)	151 (13.0%)	151 (14.3%)	153 (12.8%)
Ever or partner ever pregnant	481 (25.6%)	460 (22.9%)	521 (26.1%)	485 (24.6%)	323 (37.0%)	315 (35.9%)	344 (37.2%)	335 (39.8%)	158 (15.9%)	145 (12.6%)	177 (16.8%)	150 (12.9%)
Ever had sex	1275(67.7%)	1232 (63.0%)	1289 (68.2%)	1261(68.2%)	531 (61.0%)	510 (60.7%)	549 (61.9%)	508 (61.8%)	744 (73.5%)	722 (64.7%)	740 (73.5%)	753 (72.7%)
Used a condom last time had sex^a^	527 (40.7%)	471 (40.9%)	513 (42.7%)	513 (42.0%)	170 (33.7%)	146 (33.7%)	162 (33.2%)	155 (35.1%)	357 (45.3%)	325 (46.4%)	351 (49.6%)	358 (47.2%)
Has had multiple sex partners	593 (32.0%)	554 (28.1%)	585 (29.3%)	597 (33.1%)	183 (22.2%)	146 (19.9%)	180 (20.4%)	185 (24.6%)	410 (40.2%)	408 (34.5%)	405 (36.6%)	412 (39.7%)
Ever tested for HIV (VCT)	861 (44.0%)	828 (43.9%)	820 (42.4%)	789 (41.6%)	460 (51.7%)	438 (52.4%)	451 (48.7%)	422 (54.0%)	401 (37.5%)	390 (37.1%)	369 (37.1%)	367 (32.9%)
Believed likelihood of current HIV infection >0	411 (22.9%)	443 (24.1%)	457 (24.7%)	434 (22.4%)	168 (19.8%)	166 (21.0%)	173 (20.9%)	173 (20.8%)	243 (25.7%)	277 (26.5%)	284 (27.9%)	261 (23.8%)
Believed likelihood of future HIV infection >0	621 (33.3%)	622 (34.3%)	657 (36.8%)	607 (32.7%)	290 (36.2%)	262 (30.3%)	285 (36.7%)	277 (33.8%)	331 (30.9%)	360 (37.2%)	372 (36.9%)	330 (31.7%)
Named at least 3 ways to prevent HIV	503 (28.2%)	523 (29.6%)	538 (31.9%)	506 (29.4%)	230 (27.1%)	219 (28.1%)	228 (33.3%)	212 (29.0%)	273 (29.4%)	304 (30.7%)	310 (30.7%)	294 (30.1%)
Correctly answered 3 HIV knowledge questions	641 (34.6%)	623 (35.0%)	643 (33.8%)	630 (33.9%)	322 (38.5%)	291 (36.5%)	302 (35.2%)	294 (36.4%)	319 (31.0%)	332 (33.7%)	341 (32.6%)	336 (32.5%)
Indicated positive attitudes towards people living with HIV	269 (13.1%)	271 (13.6%)	254 (12.5%)	270 (13.7%)	126 (12.9%)	125 (15.8%)	92 (10.0%)	101 (13.3%)	143 (13.2%)	146 (12.1%)	162 (14.7%)	169 (14.8%)

Note: Figures include the Number (weighted % to take into account intensive tracking).

^a^ restricted to those who ever had sex.

Table 2 shows HSV-2 and HIV outcomes per group and by sex. Overall, none of the interventions significantly reduced HSV-2 prevalence compared to control in the full sample or among the female or male subgroups. In the logistic regressions, the adjusted odds ratios for HSV-2 among the full sample are: 0.87 (95% CI: 0.61–1.25) for condoms only, 0.94 (95% CI: 0.64–1.38) for VCT only, and 1.12 (95% CI: 0.79–1.58) for both interventions. The weighted HSV-2 prevalence at follow up was similar across groups (10.8% in control, 9.1% in condoms, 10.2% in VCT only, and 11.5% in VCT and condoms). There was also no difference when restricting to only those who had a negative HSV-2 test at baseline, and there were no significant reductions in HIV prevalence across interventions. In addition, there were also no significant differences in HSV-2 prevalence when comparing the treatment arms with each other (not shown).

**Table 2:** Impact on HSV-2 and HIV; intention to treat analysis.

	Control		Condoms Only			VCT only			VCT and Condoms		
	# of Cases (weighted %)	Inc. Rate^1^	# of Cases (weighted %)	Inc. Rate^1^	Adjusted Odds Ratio^т^ (95% CI)	# of Cases (weighted %)	Inc. Rate^1^	Adjusted Odds Ratio^т^ (95% CI)	# of Cases (weighted %)	Inc. Rate^1^	Adjusted Odds Ratio^т^ (95% CI)
***Panel A*: *All***											
HSV-2	196/1822		173/1778		0.87	173/1804		0.94	197/1769		1.12
(N = 7,173)	(10.8%)		(9.1%)		(0.61–1.25)	(10.2%)		(0.64–1.38)	(11.5%)		(0.79–1.58)
HSV-2 (Baseline test	91/1717	2.35	74/1679	1.95	0.78	70/1701	1.80	0.74	89/1661	2.36	1.21
Test Negative)	(5.5%)		(4.2%)		(0.45–1.34)	(4.2%)		(0.42–1.33)	(6.4%)		(0.72–2.04)
HIV	11/1412		11/1396		0.86	8/1479		0.82	13/1427		1.48
(N = 5,714)	(0.8%)		(0.6%)		(0.34–2.16)	(0.6%)		(0.25–2.66)	(1.1%)		(0.52–4.18)
***Panel B*: *Female***											
HSV-2	120/838		104/787		0.96	103/808		1.00	119/793		1.12
(N = 3,226)	(14.1%)		(12.9%)		(0.58–1.6)	(14.2%)		(0.59–1.7)	(14.9%)		(0.69–1.81)
HSV-2 (Baseline	55/773	3.16	40/723	2.42	0.9	41/746	2.40	0.92	56/730	3.40	1.39
Test Negative)	(7.0%)		(6.1%)		(0.41–1.96)	(6.5%)		(0.40–2.11)	(9.1%)		(0.68–2.85)
HIV (N = 2,680)	6/691		6/640		1.12	8/683		2.11	10/666		2.51
	(0.7%)		(0.7%)		(0.34–3.64)	(1.3%)		(0.64–6.98)	(1.5%)		(0.79–7.98)
***Panel C*: *Male***											
HSV-2	76/984		69/991		0.77	70/996		0.89	78/976		1.16
(N = 3,947)	(7.6%)		(5.9%)		(0.51–1.17)	(6.8%)		(0.54–1.48)	(8.6%)		(0.74–1.82)
HSV-2 (Baseline	36/944	1.69	34/956	1.59	0.7	29/955	1.34	0.59	33/931	1.55	1.15
Test Negative)	(4.1%)		(2.8%)		(0.40–1.23)	(2.3%)		(0.32–1.06)	(4.4%)		(0.58–2.28)
HIV	5/721		5/756		1.04	0/796		0.00	3/761		0.86
(N = 3,034)	(0.9%)		(0.5%)		(0.32–3.43)	(0.0%)		(0.0–0.0)	(0.6%)		(0.13–5.89)

Note

^1^: Incidence rate per 100 Person-Years.

т: Adjusted odds ratio calculated from a logistic regression. Each row corresponds to a separate regression. The following control variables were used in the regression: age categories (<19, 19–20, 20–22, >22), gender, months between baseline and follow up, secondary school enrollment in 2007, whether pregnant by 2007, and treatment in 2003. Logistics regressions include intensive tracking weights.

Table 3 presents the effects of the interventions on secondary outcomes at follow up. Respondents in both condom treatment groups were statistically significantly more likely to report receiving and using at least some free condoms than respondents in the control group (adj OR: 2.07, 95% CI: 1.62–2.64 for condoms only, and adj OR: 2.21, 95% CI: 1.76–2.77 for condoms and VCT group). Respondents in both VCT treatment arms were significantly more likely to report ever having VCT than those in the control group (adj OR: 3.54, 95% CI: 2.32–5.41 for VCT only, and adj OR: 5.52, 95% CI: 3.90–7.81 for condoms and VCT group). On other behavior outcomes, including having multiple partners in the last 6 months, using a condom during last sex, self-reported STI symptoms as well as on having basic knowledge about HIV/AIDS, there were no statistically significant differences between the control and treatment groups.

**Table 3:** Impact on secondary outcomes; intention to treat analysis.

	Control	Condoms Only		VCT only		VCT and Condoms	
	# of Cases (weighted %)	# of Cases (weighted %)	Adjusted Odds Ratio^т^ (95% CI)	# of Cases (weighted %)	Adjusted Odds Ratio^т^ (95% CI)	# of Cases (weighted %)	Adjusted Odds Ratio^т^ (95% CI)
***Panel A*: *All***							
Ever received condoms for free (in lifetime)	784/1918	1311/1870	3.85	803/1888	1.08	1308/1867	3.63
	(41.7%)	(69.5%)	(3.01–4.93)	(43.3%)	(0.86–1.35)	(68.8%)	(2.91–4.53)
Ever received condoms for free and reported using at least some of them	470/1872	763/1848	2.07	471/1833	0.96	777/1843	2.21
	(26.4%)	(40.6%)	(1.62–2.64)	(25.7%)	(0.74–1.24)	(42.5%)	(1.76–2.77)
Ever sold or gave away condoms	384/738	928/1294	2.22	385/750	0.95	935/1286	3.02
	(53.6%)	(70.5%)	(1.62–3.05)	(52.6%)	(0.69–1.31)	(76.2%)	(2.27–4.02)
Ever had VCT (in lifetime)	1525/1922	1474/1872	0.85	1798/1892	3.54	1784/1869	5.52
	(82.4%)	(79.3%)	(0.66–1.10)	(94.1%)	(2.32–5.41)	(96.1%)	(3.90–7.81)
Had VCT more than once	1160/1913	1136/1858	0.95	1382/1875	1.76	1403/1855	2.08
	(65.1%)	(62.8%)	(0.76–1.19)	(75.9%)	(1.40–2.21)	(78.4%)	(1.66–2.59)
Had VCT more than twice	774/1913	752/1858	0.98	965/1875	1.39	978/1855	1.56
	(45.2%)	(43.5%)	(0.78–1.24)	(52.6%)	(1.10–1.75)	(55.0%)	(1.25–1.95)
Currently Married	691/1923	639/1874	0.83	676/1892	0.84	638/1872	0.86
	(38.8%)	(33.1%)	(0.64–1.07)	(34.8%)	(0.64–1.09)	(34.8%)	(0.66–1.11)
Had sex in the last 6 months	1273/1923	1215/1875	0.87	1281/1890	0.95	1197/1870	0.86
	(69.2%)	(65.1%)	(0.69–1.10)	(67.6%)	(0.75–1.2)	(65.7%)	(0.69–1.07)
Number of partners in the last 6 months	0.9	0.9	0.9	0.9	0.97	0.9	0.9
			(0.73–1.11)		(0.79–1.19)		(0.74–1.09)
Total number of partners	3.1	2.9	0.95	3.1	0.91	3.6	1.07
			(0.78–1.15)		(0.75–1.11)		(0.89–1.28)
Ever used a condom	1188/1613	1218/1612	1.26	1190/1617	1.09	1202/1578	1.2
	(73%)	(77.3%)	(0.95–1.68)	(74.6%)	(0.81–1.45)	(76.5%)	(0.91–1.59)
Has had sex and did not use a condom last time	919/1822	909/1778	1.04	941/1804	1.06	898/1769	1.07
	(51.8%)	(50.4%)	(0.81–1.32)	(52.4%)	(0.84–1.35)	(52.0%)	(0.85–1.34)
Has unprotected sex with non-monogamous partner	436/1923	409/1875	0.85	431/1894	0.88	401/1873	0.86
	(25.8%)	(21.9%)	(0.65–1.11)	(23.5%)	(0.67–1.16)	(22.6%)	(0.66–1.11)
Self-reported STIs, sores, ulcers, or discharges in last 12 months	67/1922	47/1873	0.81	65/1892	1.19	45/1869	1.18
	(3.1%)	(2.4%)	(0.44–1.49)	(3.6%)	(0.72–1.99)	(3.6%)	(0.66–2.12)
Named at least 3 ways to prevent HIV	758/1818	755/1770	0.97	723/1801	0.75	706/1764	0.87
	(43.7%)	(43.9%)	(0.77–1.23)	(37.6%)	(0.6–0.95)	(41.5%)	(0.69–1.09)
Correctly answered 3 HIV knowledge questions	562/1812	539/1764	1.02	516/1796	0.89	524/1762	1.02
	(31.2%)	(31.8%)	(0.81–1.3)	(28.9%)	(0.7–1.13)	(31.9%)	(0.81–1.29)
Indicated positive attitudes towards people living with HIV (PLHIV)	301/1808	308/1766	1.03	319/1791	1.3	298/1760	1.05
	(16.7%)	(17.5%)	(0.77–1.36)	(20.7%)	(0.97–1.73)	(18%)	(0.8–1.37)
Ever or partner ever pregnant	794/1921	784/1874	0.91	811/1888	0.99	780/1868	0.95
	(44%)	(39.9%)	(0.7–1.18)	(43.1%)	(0.76–1.29)	(41.9%)	(0.74–1.22)
Number of children	0.7	0.7	0.95	0.7	1.05	0.7	0.98
			(0.75–1.21)		(0.82–1.34)		(0.78–1.23)
***Panel B*: *Female***							
Ever received condoms for free (in lifetime)	191/873	409/821	4.32	211/840	1.42	426/820	3.78
	(20%)	(50.2%)	(3.07–6.07)	(26.1%)	(1–2.02)	(47.1%)	(2.7–5.29)
Ever received condoms for free and reported using at least some of them	76/846	173/809	3.42	94/812	1.74	200/807	3.60
	(7.4%)	(20.7%)	(2.26–5.17)	(12%)	(1.1–2.73)	(21.7%)	(2.41–5.36)
Ever sold or gave away condoms	81/164	269/399	2.00	89/183	0.81	279/415	2.91
	(47.4%)	(66.2%)	(1.13–3.55)	(43.7%)	(0.44–1.51)	(72.6%)	(1.73–4.88)
Ever had VCT (in lifetime)	754/876	706/821	0.75	820/841	6.93	801/821	7.38
	(89.0%)	(84.8%)	(0.49–1.14)	(98.2%)	(4.23–11.35)	(98.2%)	(4.47–12.19)
Had VCT more than once	614/873	589/815	1.05	695/837	2.52	684/813	2.63
	(72.4%)	(71.7%)	(0.72–1.53)	(86.1%)	(1.77–3.57)	(86.5%)	(1.79–3.85)
Had VCT more than twice	441/873	423/815	1.03	531/837	1.50	513/813	1.67
	(53.3%)	(52.2%)	(0.73–1.44)	(61.9%)	(1.05–2.14)	(64.2%)	(1.18–2.37)
Number of partners in the last 6 months	0.7	0.7	1.16	0.7	0.98	0.7	1.01
			(0.83–1.63)		(0.71–1.35)		(0.73–1.38)
Total number of partners	1.5	1.5	1.07	1.5	0.98	1.6	1.24
			(0.81–1.43)		(0.72–1.34)		(0.93–1.64)
Ever used a condom	458/692	466/685	1.46	478/703	1.26	474/671	1.35
	(63%)	(71.2%)	(0.97–2.19)	(68.2%)	(0.83–1.89)	(69.6%)	(0.89–2.06)
Has had sex and did not use a condom last time	482/838	476/787	1.02	462/808	0.81	474/793	1.20
	(61.9%)	(60.0%)	(0.7–1.47)	(56.3%)	(0.57–1.16)	(64.4%)	(0.85–1.68)
Has unprotected sex with non-monogamous partner	247/877	221/823	0.85	231/842	0.85	243/822	0.92
	(33.0%)	(27.8%)	(0.59–1.22)	(29.1%)	(0.57–1.26)	(30.2%)	(0.64–1.32)
Self-reported STIs, sores, ulcers, or discharges in last 12 months	34/876	25/822	1.02	39/842	1.20	22/822	1.25
	(3.6%)	(3.5%)	(0.42–2.46)	(4.4%)	(0.63–2.3)	(4.6%)	(0.50–3.12)
Correctly answered 3 HIV knowledge questions	277/834	257/781	0.94	252/805	0.94	246/789	1.02
	(31.9%)	(31.4%)	(0.67–1.33)	(30.4%)	(0.67–1.31)	(32.9%)	(0.72–1.45)
Indicated positive attitudes towards people living with HIV (PLHIV)	124/829	139/784	1.19	145/802	1.79	134/787	1.14
	(14.5%)	(17.3%)	(0.79–1.81)	(23.4%)	(1.16–2.75)	(16.4%)	(0.74–1.75)
Ever pregnant	468/876	463/822	0.91	471/841	0.79	471/822	1.01
	(57.7%)	(53.4%)	(0.62–1.34)	(51%)	(0.53–1.17)	(57.1%)	(0.70–1.45)
Number of children	1.0	0.9	0.97	0.9	0.89	1.0	0.99
			(0.68–1.37)		(0.64–1.25)		(0.73–1.35)
***Panel C*: *Male***							
Ever received condoms for free (in lifetime)	593/1045	902/1049	3.82	592/1048	0.91	882/1047	3.65
	(60.0%)	(84.6%)	(2.66–5.49)	(57.6%)	(0.68–1.22)	(84.3%)	(2.71–4.93)
Ever received condoms for free and reported using at least some of them	394/1026	590/1039	1.81	377/1021	0.81	577/1036	1.87
	(42.4%)	(56.1%)	(1.34–2.43)	(37.1%)	(0.60–1.11)	(57.5%)	(1.42–2.46)
Ever sold or gave away condoms	303/574	659/895	2.28	296/567	1.01	656/871	2.89
	(55.3%)	(72.5%)	(1.57–3.3)	(55.9%)	(0.70–1.45)	(77.3%)	(2.07–4.04)
Ever had VCT (in lifetime)	771/1046	768/1051	0.92	978/1051	2.98	983/1048	5.19
	(76.8%)	(74.9%)	(0.68–1.24)	(90.7%)	(1.80–4.94)	(94.4%)	(3.53–7.65)
Had VCT more than once	546/1040	547/1043	0.91	687/1038	1.48	719/1042	1.88
	(58.8%)	(55.8%)	(0.69–1.2)	(67.3%)	(1.11–1.99)	(72.1%)	(1.45–2.43)
Had VCT more than twice	333/1040	329/1043	0.96	434/1038	1.33	465/1042	1.51
	(38.4%)	(36.6%)	(0.70–1.31)	(44.6%)	(0.98–1.80)	(47.6%)	(1.14–2.00)
Number of partners in the last 6 months	1.1	1.0	0.79	1.1	0.99	1.0	0.86
			(0.61–1.03)		(0.76–1.29)		(0.68–1.10)
Total number of partners	4.4	4.0	0.86	4.4	0.88	5.0	0.96
			(0.66–1.12)		(0.67–1.14)		(0.76–1.23)
Ever used a condom	730/921	752/927	1.09	712/914	0.93	728/907	1.03
	(80.8%)	(82%)	(0.73–1.62)	(79.7%)	(0.62–1.4)	(81.2%)	(0.7–1.5)
Has had sex and did not use a condom last time	437/984	433/991	1.06	479/996	1.35	424/976	1.05
	(42.9%)	(42.5%)	(0.77–1.46)	(49.1%)	(0.98–1.86)	(42.7%)	(0.77–1.43)
Has unprotected sex with non-monogamous partner	189/1046	188/1052	0.88	200/1052	0.94	158/1051	0.82
	(19.7%)	(17.2%)	(0.62–1.26)	(18.8%)	(0.65–1.37)	(16.5%)	(0.57–1.17)
Self-reported STIs, sores, ulcers, or discharges in last 12 months	33/1046	22/1051	0.59	26/1050	1.09	23/1047	1.10
	(2.7%)	(1.6%)	(0.33–1.06)	(2.9%)	(0.48–2.49)	(3.0%)	(0.52–2.31)
Correctly answered 3 HIV knowledge questions	285/978	282/983	1.08	264/991	0.85	278/973	1.00
	(30.7%)	(31.9%)	(0.79–1.49)	(27.7%)	(0.61–1.18)	(31.2%)	(0.74–1.35)
Indicated positive attitudes towards people living with HIV (PLHIV)	177/979	169/982	0.89	174/989	0.98	164/973	0.97
	(18.8%)	(17.7%)	(0.61–1.30)	(18.5%)	(0.68–1.41)	(19.1%)	(0.69–1.36)
Partner ever pregnant	326/1045	321/1052	0.92	341/1047	1.24	309/1046	0.93
	(32.1%)	(29%)	(0.65–1.31)	(36.4%)	(0.86–1.79)	(29.8%)	(0.65–1.32)
Number of children	0.5	0.4	0.93	0.6	1.25	0.5	0.98
			(0.67–1.29)		(0.89–1.75)		(0.71–1.37)

Note

т: Adjusted odds ratio calculated from a logistic regression. Each row corresponds to a separate regression. The following control variables were used in the regression: age categories (<19, 19–20, 20–22, >22), sex, months between baseline and follow up, secondary school enrollment in 2007, whether pregnant by 2007, and treatment in 2003. Logistics regressions include intensive tracking weights.

Table 3 also includes two measures related to childbearing, ever being pregnant or partner ever pregnant and the number of children. Again, there are no statistically significant differences for either across treatment groups (ex. for ever being pregnant or partner ever pregnant: adj OR: 0.91, 95% CI: 0.7–1.18 for condoms only; adj OR: 0.99, 95% CI: 0.76–1.29 for VCT; adj OR: 0.95, 95% CI: 0.74–1.22 for condoms and VCT).

Table 4 presents the heterogeneity analysis, comparing impact of the intervention on HSV-2 prevalence, among those who had started childbearing at baseline and among those who had not (Panel A and Panel B). It also presents the heterogeneity analysis among those who believed their likelihood of HIV infection at baseline was zero or greater than zero (Panel C and Panel D). As can bee seen, there are no statistically significant impacts of the interventions on any of the subgroups. In addition, there are no differential effects by the initial 2003 treatment arms of the Duflo, Dupas and Kremer study [30] (not shown).

**Table 4:** Impact on HSV-2 by childbearing status and beliefs on HIV.

	Control	Condoms Only		VCT only		VCT and Condoms	
	# of Cases (weighted %)	# of Cases (weighted %)	Adjusted Odds Ratio^т^ (95% CI)	# of Cases (weighted %)	Adjusted Odds Ratio^т^ (95% CI)	# of Cases (weighted %)	Adjusted Odds Ratio^т^ (95% CI)
***Panel A*: *Started childbearing at baseline***							
HSV-2 full sample	97/464	75/436	0.89	84/507	0.91	98/463	1.01
	(19.3%)	(17%)	(0.54–1.45)	(17.8%)	(0.55–1.51)	(19.9%)	(0.64–1.57)
HSV-2 only female	69/318	60/302	1.04	61/333	0.89	79/323	1.20
	(19.9%)	(19.3%)	(0.57–1.89)	(18.5%)	(0.49–1.61)	(23.4%)	(0.68–2.14)
HSV-2 only male	28/146	15/134	0.59	23/174	0.85	19/140	0.66
	(17.0%)	(11.7%)	(0.26–1.38)	(16.1%)	(0.37–1.95)	(11.6%)	(0.31–1.38)
***Panel B*: *Not started childbearing at baseline***							
HSV-2 full sample	99/1358	98/1342	0.86	89/1297	0.94	99/1306	1.18
	(7.7%)	(6.6%)	(0.53–1.39)	(7.3%)	(0.54–1.62)	(8.8%)	(0.72–1.94)
HSV-2 only female	51/520	44/485	0.96	42/475	1.16	40/470	0.98
	(10.5%)	(9.3%)	(0.43–2.14)	(11.5%)	(0.47–2.84)	(9.5%)	(0.43–2.22)
HSV-2 only male	48/838	54/857	0.85	47/822	0.85	59/836	1.46
	(5.8%)	(4.9%)	(0.53–1.36)	(4.8%)	(0.51–1.43)	(8.2%)	(0.86–2.49)
***Panel C*: *Believed likelihood of current HIV infection >0 at baseline***							
HSV-2 full sample	56/387	52/416	0.80	37/433	0.91	53/405	1.10
	(11.4%)	(9.0%)	(0.49–1.28)	(9.7%)	(0.45–1.83)	(12.5%)	(0.62–1.95)
HSV-2 only female	32/162	29/156	0.82	21/167	0.75	30/168	0.87
	(15.9%)	(12.7%)	(0.41–1.62)	(11.6%)	(0.36–1.54)	(14.4%)	(0.42–1.8)
HSV-2 only male	24/225	23/260	0.78	16/266 (	1.12	23/237	1.23
	(8.3%)	(6.6%)	(0.40–1.53)	8.4%)	(0.36–3.43)	(10.1%)	(0.52–2.88)
***Panel D*: *Believed likelihood of current HIV infection = 0 at baseline***							
HSV-2 full sample	140/1435	121/1362	0.90	136/1371	0.95	144/1364	1.14
	(10.6%)	(9.1%)	(0.58–1.4)	(10.4%)	(0.61–1.49)	(11.2%)	(0.76–1.72)
HSV-2 only female	88/676	75/631	1.01	82/641	1.05	89/625	1.21
	(13.7%)	(12.9%)	(0.55–1.86)	(14.9%)	(0.55–1.99)	(15.1%)	(0.69–2.13)
HSV-2 only male	52/759	46/731	0.76	54/730	0.82	55/739	1.12
	(7.4%)	(5.6%)	(0.45–1.27)	(6.2%)	(0.49–1.36)	(8.1%)	(0.67–1.89)

Note

т: Adjusted odds ratio calculated from a logistic regression. The following control variables were used in the regression: age categories (<19, 19–20, 20–22, >22), gender, months between baseline and follow up, secondary school enrollment in 2007, whether pregnant by 2007, and treatment in 2003. Logistics regressions include intensive tracking weights.

## Discussion

The trial demonstrates no significant impacts of a community-based VCT program, distribution of free male condoms, or both programs together on HSV-2 prevalence, 2 years post-intervention among youth in Western Kenya. While the adjusted odds ratios for the condoms only and VCT only treatments are in the expected, negative direction, the combined treatment group produces an unexpected positive indicator of the treatment effect, but none of these effects are significantly different from zero. Similarly, there was no impact of any of the interventions on HSV-2 incidence or HIV prevalence, though the study was not powered to detect a difference in HIV prevalence. This study also found no significant impact on most self-reported behavioral outcomes and childbearing. The results thus suggest that neither program alone or together led to significant behavior change within our sample.

Several other studies have assessed the impact of VCT on sexual behaviors [10–19], but only a few have assessed biological endpoints [18,19]. Most reported effects have been among those testing positive for HIV [11–14]. For example, Thornton found significant self-reported behavioral changes in individuals who tested positive but no effects for those who tested negative [14]. A meta-analysis synthesizing results across 17 studies reported no significant difference in HIV incidence, and STI prevalence or incidence among participants receiving VCT, but found reductions in number of sexual partners, and increased condom use among those who received a positive test result [11]. Our findings also show no significant change in biological endpoints, which is consistent with these studies. However, in this study, it was not possible to assess impacts among the subgroup testing positive for HIV given the very low HIV prevalence in the sample. Unlike some previous studies, this trial does not find change in reported risky sexual behaviors, but our study population comprised youth aged 17 to 24 years, a population not been well studied with respect to VCT. The results are also consistent with a more recent trial of a multi-component HIV prevention program which included community-based VCT conducted in four sites in Africa and in Thailand [19]. It reported significant reductions in HIV incidence among certain subgroups including older women but also did not reduce HIV incidence among young people aged 18–24 years [19].

Importantly, our study findings demonstrate statistically significant increased odds of ever having VCT in both the VCT arm the VCT plus condoms arm compared to the control arm (adjusted odds ratios of 3.54 and 5.52 respectively) indicating increased HIV testing among this youth population. This is an important finding given that recent estimates suggest only 46% of women and 53% of men in Kenya have ever been tested for HIV [46] and in light of the numerous barriers that reduce access to HIV testing for youth [21]. As accessible VCT services are considered to be essential for any HIV prevention programming, our results suggest that community-based VCT can help increase access to HIV testing even in settings where VCT services are widely available.

Finally, we also assessed whether intervention impacts varied depending on individuals’ perceptions of their own HIV risk at baseline. Previous research by Gong in two major Kenyan and Tanzanian cities reported that HIV test results influence behavior primarily when they provide unexpected information [47]. In that study, individuals who expected an HIV positive result but tested negative were more likely to decrease risky sexual behaviors. In our study, we found no significant change in biological or behavior outcomes among individuals whose perceived HIV risk was low or high. We also see no evidence of disinhibition or increased risk behaviors in those testing negative in this population.

This study has several limitations. One serious caveat to the null VCT results is the high uptake of VCT across all groups at baseline, and within the control group over the period between baseline and follow up. At baseline, 44% of the control group had been tested for HIV, while at follow up 82.4% (89% of females and 76.8% of males) had participated in VCT, as it is available for free at local health clinics and is a standard component of antenatal care. In addition, there were several large-scale HIV testing campaigns in the area that were initiated during the study follow up period including the USAID funded APHIA II [48]. The high VCT uptake in the non-VCT groups during the study period potentially attenuates treatment effects, and this should be taken into consideration when interpreting the findings. Future studies assessing VCT uptake and impact should be designed among populations or in contexts with more limited access to HIV testing.

Uptake is also an important consideration to the null condom results, but in the other direction; overall approximately 70% of those sampled to receive condoms reported receiving condoms at the follow up visit. However, there was variation by sex as about 50% of females compared to 85% of males in the condoms treatments reported receiving the free condoms. Conditional on reporting receipt of free condoms, only 21% of females and 56% of males reported ever using them and more than 65% of both males and females report selling or giving away at least some of the condoms. In addition to the low uptake and use, this also suggests spillover effects which potentially attenuate the treatment effects as measured. It is worthwhile to note the odds of using the free condoms was still significantly higher in the condom arms than in the control (adjusted odds ratios of 2.07 in the condoms only group and 2.21 in the condoms and VCT group) suggesting that the interventions helped address factors limiting youth access to and use of condoms in this setting.

Finally, another potential limitation is that the sample population participated in a previous HIV prevention trial which could have influenced the results. However, our findings demonstrate no differential effects by treatment arm from the previous study suggesting that the prior treatment assignment did not influence study findings. Participation in the initial trial does reduce the external validity of the study, but as HIV prevention programming is being scaled up, there are many contexts where populations are exposed to multiple types of HIV interventions.

In conclusion, this study suggests that in contexts where VCT and condoms are widely available, community-based VCT campaigns and direct condom distribution may be unlikely on their own to significantly reduce the prevalence of HSV-2 among youth with at least some primary education. However, in such settings, community-based VCT can still significantly increase HIV testing among youth, who are often hard to reach and face numerous barriers to accessing HIV prevention programs. Increasing access to male condoms can increase condom use among youth, but is unlikely to affect childbearing in this population. Additional complementary HIV prevention interventions targeting young people, including those aimed at increasing demand side challenges, are needed.

## Supporting information

S1 File CONSORT checklist. (DOC)

S2 File Study protocol. (DOC)

## References

[1] UNAIDS. Fact Sheet 2018. Available from: http://www.unaids.org/en/resources/fact-sheet [Cited 11 June 2019].

[2] UNAIDS. AIDS Info New: HIV Infections. Available from http://aidsinfo.unaids.org/ [Cited Jun 11 2019].

[3] UNAIDS. Women and HIV: A Spotlight on Adolescent Girls and Young Women. 2019.

[4] UNAIDS. UNAIDS Gap Report 2014. 2015.

[5] Oberzaucher N, Baggaley R. HIV Voluntary Counselling and Testing: A Gateway to Prevention and Care. Five case studies related to the prevention of mother-to-child transmission of HIV, tuberculosis, young people, and reaching general population groups 2002 UNAIDS.

[6] De Cock KM, Marum E, Mbori-Ngacha D. A serostatus-based approach to HIV/AIDS prevention and care in Africa. Lancet. 2003; 362(9398): 1847–1849. 10.1016/S0140-6736(03)14906-9 14654325

[7] WHO. Scaling-up HIV testing and counselling services: a toolkit for program managers. 2004. Available from: http://www.who.int/hiv/pub/vct/toolkit/en/index.html [Cited 21 August 2017].

[8] Arthur G, Nduba V, Forsythe S, Mutemi R, Odhiambo J, Gilks C. Behaviour change in clients of health centre-based voluntary HIV counselling and testing services in Kenya. Sex Transm Infect. 2007; 83(7):541–6. 10.1136/sti.2007.026732 17991688 PMC2598642

[9] Matambo R, Dauya E, Mutswanga J, Makanza E, Chandiwana S, Mason PR, et al Voluntary counseling and testing by nurse counselors: what is the role of routine repeated testing after a negative result? Clin Infect Dis. 2006; 42(4):569–71 10.1086/499954 16421803

[10] Cawley C, Wringe A, Slaymaker E, Todd J, Michael D, Kumugola Y, et al The impact of voluntary counselling and testing services on sexual behaviour change and HIV incidence: observations from a cohort study in rural Tanzania. BMC infectious diseases 2014; 14(159). 10.1186/1471-2334-14-159 PMC3994406 24655360

[11] Fonner VA, Denison J, Kennedy CE, O'Reilly K, Sweat M. Voluntary counseling and testing (VCT) for changing HIV-related risk behavior in developing countries. The Cochrane Library 2012 10.1002/14651858.CD010274 PMC3931252 22972050

[12] Denison JA, O'Reilly KR, Schmid GP, Kennedy CE, Sweat MD. HIV voluntary counseling and testing and behavioral risk reduction in developing countries: a meta-analysis, 1990–2005. AIDS and Behavior. 2008; 12(3):363–373. 10.1007/s10461-007-9349-x 18161018

[13] The Voluntary HIV-1 Counseling and Testing Efficacy Study Group. Efficacy of voluntary HIV-1 counselling and testing in individuals and couples in Kenya, Tanzania, and Trinidad: a randomised trial. Lancet. 2000; 356(9224):103–112. 10963246

[14] Thornton RL. The Demand for, and Impact of, Learning HIV Status. American Economic Review. 2008; 98(5):1829–63. 10.1257/aer.98.5.1829 21687831 PMC3115776

[15] Matovu JKB, Gray RH, Makumbi F, Wawer MJ, Serwadda D, Kigozi G, et al Voluntary HIV counseling and testing acceptance, sexual risk behavior and HIV incidence in Rakai, Uganda. AIDS. 2005; 19(5):503–511. 10.1097/01.aids.0000162339.43310.33 15764856

[16] Turner AN, Miller WC, Padian NS, Kaufman JS, Behets FM, Chipato T, et al Unprotected sex following HIV testing among women in Uganda and Zimbabwe: short- and long-term comparisons with pre-test behaviour. International Journal of Epidemiology. 2009; 38(4):997–1007. 10.1093/ije/dyp171 19349481 PMC2720394

[17] Sherr L, Lopman B, Kakowa M, Dube S, Chawira G, Nyamukapa C, et al Voluntary counselling and testing: uptake, impact on sexual behaviour, and HIV incidence in a rural Zimbabwean cohort. AIDS. 2007; 21(7):851–860. 10.1097/QAD.0b013e32805e8711 17415040

[18] Corbett EL, Makamure B, Cheung YB, Dauya E, Matambo R, Bandason T, et al HIV incidence during a cluster-randomized trial of two strategies providing voluntary counselling and testing at the workplace, Zimbabwe. AIDS. 2007; 21(4):483–9. 10.1097/QAD.0b013e3280115402 17301567

[19] Coates TJ, Kulich M, Celentano DD, Zelaya CE, Chariyalertsak S, Chingono A, et al Effect of community-based voluntary counselling and testing on HIV incidence and social and behavioural outcomes (NIMH Project Accept; HPTN 043): a cluster-randomised trial. Lancet Global Health. 2014;2(5): E267–77. 10.1016/S2214-109X(14)70032-4 25103167 PMC4131207

[20] World Health Organization. HIV and adolescents: guidance for HIV testing and counselling and care for adolescents living with HIV: recommendations for a public health approach and considerations for policy-makers and managers. 2013. 25032477

[21] Govindasamy D, Ferrand RA, Wilmore SM, Ford N, Ahmed S, Afnan‐Holmes H, et al Uptake and yield of HIV testing and counselling among children and adolescents in sub‐Saharan Africa: a systematic review. Journal of the International AIDS Society. 2015;18(1):20182. 26471265 10.7448/IAS.18.1.20182 PMC4607700

[22] Musheke M, Ntalasha H, Gari S, Mckenzie O, Bond V, Martin-Hilber A, et al A systematic review of qualitative findings on factors enabling and deterring uptake of HIV testing in Sub-Saharan Africa. BMC Public Health. 2013; 13, 220 10.1186/1471-2458-13-220 23497196 PMC3610106

[23] Idele P, Gillespie A, Porth T, Suzuki C, Mahy M, Kasedde S, et al Epidemiology of HIV and AIDS among adolescents: current status, inequities, and data gaps. J Acquir Immune Defic Syndr. 2014; 66: p. S144–S153 10.1097/QAI.0000000000000176 24918590

[24] Oginni A, Obianwu O, Adebajo S. Socio-demographic Factors Associated with Uptake of HIV Counseling and Testing (HCT) among Nigerian Youth. AIDS Research And Human Retroviruses. 2014; 30 (S1) A113–A113 10.1089/aid.2014.5216.abstract

[25] Nall A, Chenneville T, Rodriguez LM, O’Brien JL. Factors Affecting HIV Testing among Youth in Kenya. International journal of environmental research and public health. 2019;16(8):1450. 10.3390/ijerph16081450 PMC6517959 31022872

[26] Asaolu IO, Gunn JK, Center KE, Koss MP, Iwelunmor JI, Ehiri JE. Predictors of HIV testing among youth in sub-Saharan Africa: a cross-sectional study. PloS one. 2016;11(10):e0164052 10.1371/journal.pone.0164052 27706252 PMC5051677

[27] Sabapathy K, Van den Bergh R, Fidler S, Hayes R, Ford N. Uptake of home-based voluntary HIV testing in sub-Saharan Africa: a systematic review and meta-analysis. PLoS Med. 2012;9(12):e1001351 10.1371/journal.pmed.1001351 23226107 PMC3514284

[28] Grabbe KL, Menzies N, Taegtmeyer M, Emukule G, Angala P, Mwega I, et al Increasing access to HIV counseling and testing through mobile services in Kenya: strategies, utilization, and cost-effectiveness. J Acquir Immune Defic Syndr. 2010;54(3): 317–23. 10.1097/QAI.0b013e3181ced126 20453819 PMC3225204

[29] Cremin I, Nyamukapa C, Sherr L, Hallett TB, Chawira G, Cauchemez S, et al Patterns of self-reported behaviour change associated with receiving voluntary counselling and testing in a longitudinal study from Manicaland, Zimbabwe. AIDS and Behavior. 2010; 14(3):708–15. 10.1007/s10461-009-9592-4 19623481 PMC2865634

[30] Pant Pai N, Sharma J, Shivkumar S, Pillay S, Vadnais C, Joseph L, et al Supervised and unsupervised self-testing for HIV in high- and low-risk populations: a systematic review. PLoS Med. 2013;10(4):e1001414 10.1371/journal.pmed.1001414 23565066 PMC3614510

[31] Salazar-Austin N, Kulich M, Chingono A, Chariyalertsak S, Srithanaviboonchai K, Gray G, et al Age-Related Differences in Socio-demographic and Behavioral Determinants of HIV Testing and Counseling in HPTN 043/NIMH Project Accept. AIDS and Behavior. 2018;22(2):569–79. 10.1007/s10461-017-1807-5 28589504 PMC5718984

[32] Chandra-Mouli V, McCarraher DR, Phillips SJ, Williamson NE, Hainsworth G. Contraception for adolescents in low and middle income countries: needs, barriers, and access. Reproductive health. 2014;11(1):1 10.1186/1742-4755-11-1 24383405 PMC3882494

[33] Foss AM, Hossain M, Vickerman PT, Watts CH. A systematic review of published evidence on intervention impact on condom use in sub-Saharan Africa and Asia. Sexually Transmitted Infections. 2007; 83:510–16. 10.1136/sti.2007.027144 17932124 PMC2598651

[34] O’Reilly KR, Fonner VA, Kennedy CE, Sweat MD. Free condom distribution: what we don’t know may hurt us. AIDS and Behavior. 2014; 18(11):2169–2171. 10.1007/s10461-014-0742-y 24633741 PMC4729184

[35] Cohen J, Dupas P. Free Distribution or Cost-Sharing? Evidence from a Randomized Malaria Prevention Experiment. Quarterly Journal of Economics. 2010; 125(1):1–45.

[36] Dupas P. Short-Run Subsidies and Long-Run Adoption of New Health Products: Evidence from a field experiment. Econometrica. 2014; 82(1):197–228. 10.3982/ECTA9508 25308977 PMC4193678

[37] Dupas P, Hoffmann V, Kremer M, Zwane A. Targeting health subsidies through a non-price mechanism: A randomized controlled trial in Kenya. Science. 2016; 353(6302):889–895. 10.1126/science.aaf6288 27563091 PMC5003414

[38] Papo JK, Bauni EK, Sanders EJ, Brocklehurst P, Jaffe HW. Exploring the condom gap: is supply or demand the limiting factor–condom access and use in an urban and a rural setting in Kilifi district, Kenya. AIDS. 2016;25(2):247–55. 10.1097/QAD.0b013e328341b9b8 21150559

[39] National AIDS and STI Control Programme, Kenya (NASCOP). 2007 Kenya AIDS Indicator Survey Final Report. 2009.

[40] Mugo N, Dadabhai SS, Bunnell R, Williamson J, Bennett E, Baya I, et al Prevalence of herpes simplex virus type 2 infection, human immunodeficiency virus/herpes simplex virus type 2 coinfection, and associated risk factors in a national, population-based survey in Kenya. Sexually transmitted diseases. 2011;38(11):1059–66. 10.1097/OLQ.0b013e31822e60b6 21992985

[41] NASCOP. Kenya AIDS Indicator Survey 2012: Final Report. 2014. 10.1097/QAI.0000000000000152 24732813

[42] Duflo E, Dupas P, Kremer M. Education, HIV, and Early Fertility: Experimental Evidence from Kenya. American Economic Review. 2015; 105(9):2257–97. 10.1257/aer.20121607 PMC4624413 26523067

[43] Kenya National Bureau of Statistics and ICF Macro. Kenya Demographic and Health Survey 2008–09. 2010 Calverton, Maryland: Kenya National Bureau of Statistics and ICF Macro.

[44] NASCOP. Guidelines for HIV Testing and Counselling in Kenya. 2008.

[45] Behling J, Chan AK, Zeh C, Nekesa C, Heinzerling L. Evaluating HIV prevention programs: herpes simplex virus type 2 antibodies as biomarker for sexual risk behavior in young adults in resource-poor countries. PloS one. 2015;10(5):e0128370 10.1371/journal.pone.0128370 26010772 PMC4444314

[46] Kenya National Bureau of Statistics; Ministry of Health/Kenya; National AIDS Control Council/Kenya; Kenya Medical Research Institute; National Council for Population and Development/Kenya; ICF International. Kenya Demographic and Health Survey 2014. 2015. Available online: https://dhsprogram.com/pubs/pdf/fr308/fr308.pdf

[47] Gong E. HIV Testing and risky sexual behavior. The Economic Journal. 2015; 125 (582):32–60.

[48] PATH. APHIA II Western Province. Best Practices and Promising Interventions. March 2011.

