# Supplementary material for: HIV prevention among youth: A randomized controlled trial of voluntary counseling and testing for HIV and male condom distribution in rural Kenya
Source: PLoS One. 2019 Jul 30;14(7):e0219535. doi: 10.1371/journal.pone.0219535 (PMC6667138; doi:10.1371/journal.pone.0219535)
Supplement: S2 File — (DOC) [file pone.0219535.s002.doc]

|  | **Massachusetts Institute of**  **Technology**  Committee on the Use of  Humans as Experimental Subjects | **Application #**  (assigned by  COUHES) | 08090028  73 |
| --- | --- | --- | --- |
|  |  | **Date** | **UPDATED July 2016** |

**APPLICATION FOR APPROVAL TO USE HUMANS AS EXPERIMENTAL SUBJECTS (STANDARD FORM)**


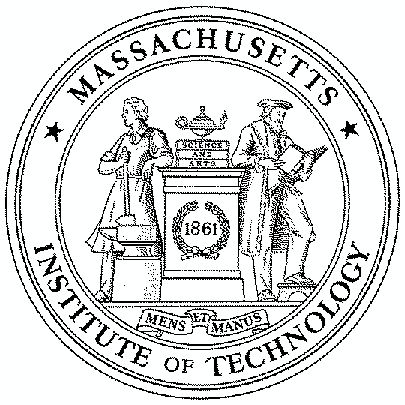


*Please answer every question. Positive answers should be amplified with details. You may mark N/A where the question does not pertain to your application. Any incomplete application will be rejected and returned for completion.* ***A completed CHECKLIST FOR STANDARD APPLICATION FORM must accompany this application.***

**I. BASIC INFORMATION**

| **1. Title of Study** | | |
| --- | --- | --- |
| Education and HIV/AIDS in Western Kenya | | |
| **2. Principal Investigator** | | |
| Name: Esther Duflo | Building and Room #: E52-544 | |
| Title: Professor of Economics | Email: [eduflo@mit.edu](mailto:eduflo@mit.edu) | |
| Department: Department of Economics | Phone: 617.258.7013 | |
| **3. Associated Investigator(s)** | | |
| Name: Pascaline Dupas, Michael Kremer,  and Vandana Sharma | Email: [pdupas@stanford.edu,](mailto:pdupas@ucla.edu)  [mkremer@fas.harvard.edu,](mailto:mkremer@fas.harvard.edu) and [vsharma@jhsph.edu](mailto:vsharma@jhsph.edu) | |
| Title: Assistant Professor of Economics,  Gates Professor of Developing Countries and Economics, and Coordinator for Public Health Studies | Phone:   | (650) 725 1870 | | --- |   , 617.495.9145, and  443.703.8547 | |
| Affiliation: Stanford University, Harvard University, and J-PAL | | |
| **4. Collaborating Institutions.** *If you are collaborating with another institution(s) then you must obtain approval from that institution’s institutional review board, and forward copies of the approval to*  *COUHES)* | | |
| KEMRI | | |
| **5. Location of Research.** *If at MIT please indicate where on campus. If you plan to use the facilities of the Clinical Research Center you will need to obtain the approval of the CRC Advisory Committee. You*  *may use this form for simultaneous submission to the CRC Advisory Committee.* | | |
| The evaluation will take place in the districts of Busia, Bungoma, and Butere-Mumias in  Western Kenya, and will be based out of the offices of Innovations for Poverty Action  (IPA), Busia, Kenya | | |
| **6. Funding.** *If the research is funded by an outside sponsor, please enclose one copy of the research proposal with your application. A draft of the research proposal is acceptable.* | | |
| Source: MacArthur Foundation | | Contract or Grant Title: HIV/AIDS  education in Kenya |
| Contract or Grant #: #04-203595 | | OSP #: 3978600, 6914789, and  6915228 |

*APPLICATION FOR APPROVAL TO USE HUMANS AS EXPERIMENTAL SUBJECTS (STANDARD FORM) – revised 9/25/2007)*- 1 -

| **7. Human Subjects Training**. *All study* personnel **MUST** *take and pass a training course on human subjects research. MIT has a web-based course that can be accessed from the main menu of the*  *COUHES web site. COUHES may accept proof of training from some other institutions. List the names of all study personnel and indicate if they have taken a human subjects training course.* | |
| --- | --- |
| Esther Duflo, Yes; Pascaline Dupas, Yes; Michael Kremer, Yes; Vandana Sharma, Yes | |
| **8. Anticipated Dates of Research** | |
| Start Date: September 31,  2008 | Completion Date: December 31, 2018 |

**II. STUDY INFORMATION**

**1. Purpose of Study.** *Please provide a concise statement of the background, nature and reasons for the proposed study. Use non-technical language that can be understood by non-scientist members of COUHES.* Education has been called a “social vaccine” for AIDS. Children aged 5 to 14 years have been referred to as a “Window of Hope,” both because they have low HIV infection rates and because their sexual behaviors are not yet established and may be more easily

molded. In Africa, the majority of children now attend some primary school, which makes schools a strategic forum for targeted HIV prevention education. There is, however, considerable debate on whether scalable school-based HIV/AIDS education programs can be effective in limiting the spread of HIV/AIDS among youth. Although many countries have incorporated HIV/AIDS education in their school curricula, limited rigorous evidence to answer questions about the effectiveness of these programs is available.

Between 2003 and 2006, the non-profit organization International Child Support (ICS) ran a large-scale AIDS prevention project in primary schools in the Western Province of Kenya. The project included 4 components: (1) The Teacher Training Program, which provided in-service training for primary school teachers to enhance the delivery of the national HIV/AIDS education curriculum; (2) The Debates on Condoms and Essay Writing Program, which promoted student-structured debates on the role of condoms and essay competitions on how they can protect themselves from HIV; (3) The Sugar Daddy Awareness Campaign, which provided adolescents with information about variation in HIV prevalence (risk) by age and sex, with the aim of reducing incidence of cross- generational sex, which exposes girls to higher HIV risk; and (4) The Reduced Cost of Education Program, which provided 20,000 free school uniforms (uniforms were distributed to children enrolled in grade 6 in year 2003, and to the same children after 18 months, when they were in grade 7), with the aim of helping youth stay in school longer. In total, 328 schools (with 70,000 students in grades 5 to 8) participated in the ICS project. All schools received the national AIDS education program, and were randomly selected to participate in one or more of the four components of the ICS AIDS prevention programs.

The random assignment of schools to various intervention groups offers a unique opportunity to rigorously measure the impact of each component of the ICS project. Follow-up data collected by ICS suggests that reducing the cost of education was an effective way to reduce teenage pregnancy rates. Providing students with information on relative infection rates (risk) led to a dramatic reduction in pregnancy and relationships

*APPLICATION FOR APPROVAL TO USE HUMANS AS EXPERIMENTAL SUBJECTS (STANDARD FORM) – revised 9/25/2007)*

- 2 -

with older partners. More interactive approaches to learning about HIV/AIDS (structured discussions of condoms and essays on prevention) impacted self-reported behavior (data on childbearing is not yet available for the cohort affected by that component). Training teachers did not lead to changes in childbearing rates, but increased the rate of marriage of female students who started childbearing (with the consequences for HIV/AIDS infection unclear).

In order to measure actual program impacts on the risk of HIV transmission, we are seeking approval to conduct a biomarker follow-up study which will determine HIV and Herpes Simplex Virus-2 (HSV-2; a virus which is usually acquired through sexual contact and can cause genital ulcer disease) prevalence among a large fraction of the students enrolled in the schools participating in the ICS program. The follow up study protocol has been designed based on the results of a small pilot study conducted between May and September 2007 which found prevalence of HSV-2 to be >10% but prevalence of other sexually transmitted infections (STIs) to be significantly lower (Chlamydia, Gonorrhea and Trichomonas Vaginalis were all <5%).

The biomarker follow-up study will involve several components. Specifically, during the

1st follow-up period commencing in September 2008, blood from all students who were enrolled in grade 6 in 2003 in participating schools and who provide written, informed consent, will be drawn and tested for HSV-2. In addition, HIV testing will be performed on the same blood samples from those respondents who provide informed consent for anonymous linked HIV testing.

We are also seeking approval under the same protocol, to examine the effectiveness of additional HIV/AIDS prevention strategies. Specifically, our biomarker follow-up study will also measure the impact of Voluntary Counseling and Testing for HIV (VCT) and provision of free condoms for two years on sexual behavior and the risk of HIV transmission. Students in our study population will be randomized to one of 4 intervention groups: 1) VCT only, 2) VCT plus free 2 year supply of condoms, 3) Free 2 year supply of condoms only, or 4) Control. These interventions will be offered during the first follow-up period and their impact will be measured during a second wave of HSV-2 testing beginning in 2010, approximately 2 years after administration of the first HSV-2 tests. During this second follow-up period, we will also offer VCT to all study participants.

A third component of this study will focus on the impact of maternal education on child health. While previous observational studies suggest an association between maternal education and child health outcomes including child nutritional status, there is continued debate as to whether this association is causal. Our study will investigate the impact of the Reduced Costs of Education Program, which aimed to keep girls in school longer, on several child health outcomes including the prevalence of anemia, stunting, wasting and underweight in children under 5 years.

**2. Study Protocol.** *For* ***biomedical, engineering and related research****, please provide an outline of the actual experiments to be performed. Where applicable, provide a detailed description of the experimental devices or procedures to be used, detailed information on the exact dosages of drugs or*

*APPLICATION FOR APPROVAL TO USE HUMANS AS EXPERIMENTAL SUBJECTS (STANDARD FORM) – revised 9/25/2007)*

- 3 -

*chemicals to be used, total quantity of blood samples to be used, and descriptions of special diets.*

*For applications in the* ***social sciences, management and other non-biomedical disciplines***

*please provide a detailed description of your proposed study. Where applicable, include copies of any*

*questionnaires or standardized tests you plan to incorporate into your study. If your study involves interviews please submit an outline indicating the types of questions you will include.*

*You should provide sufficient information for effective review by non-scientist members of*

*COUHES. Define all abbreviations and use simple words. Unless justification is provided this part of the*

*application must not exceed 5 pages.*

*Attaching sections of a grant application is not an acceptable substitute.*

The biomarkers follow-up study will be implemented by Innovations for Poverty Action, Kenya, the Kenyan affiliate of an American NGO which has extensive experience collecting data in the area. Dr. Vandana Sharma (MD, MPH) will be advising on and supervising the medical and laboratory components of this study.

Study Sample

The study will follow the cohort of approximately 20,000 students who were enrolled in grade 6 in 2003 at the onset of the ICS program. These students, who were 12 to 16 years old in 2003, will be 17 to 21 years old in 2008 at the start of the biomarkers follow-up study.

Methodology:

a) Tracking of Study Population

We will collect information on the location of respondents by visiting their former primary schools and interviewing pupils. The information collected at school will help us find the targeted respondents at their current schools or at home.

b) Survey Administration

All respondents will be asked to participate in a voluntary survey. The survey will consist of several modules and will be administered to consenting respondents during home

visits. First, respondents will be asked to answer a Knowledge, Attitudes and Skills survey (KAS) that will include questions regarding HIV prevention and attitudes towards the disease and people living with AIDS. Respondents will also be asked to answer a Behavioral Follow-up survey that will include questions on sexual behavior, past and current sexual partners, marriage, and fertility. The third module will include questions relating to socio-economic variables such as education and income, as well as general attitudes and perceptions.

c) HSV-2 testing

For HSV-2 testing, respondents will be invited to attend a mobile clinic set up near their home, and will be reimbursed for travel costs associated with attending this clinic. Respondents will be informed about the procedure and will be given the opportunity to

*APPLICATION FOR APPROVAL TO USE HUMANS AS EXPERIMENTAL SUBJECTS (STANDARD FORM) – revised 9/25/2007)*

- 4 -

give consent or to decline. At the mobile clinic, experienced nurses or phlebotomists will draw blood for HSV-2 testing from those respondents who give written consent for this procedure (approximately 5ml from the cubital vein). The blood will then be stored and sent to a certified laboratory for HSV-2 ELISA testing. We will also seek informed consent to anonymously test the same blood sample for HIV (see below). Participants who test positive for HSV-2 will be referred to a nearby STI clinic for consultation and treatment as needed based on Kenyan Ministry of Health guidelines.

d) VCT

VCT will be offered during the first follow up period, to all respondents randomized to one of the VCT intervention groups (VCT only or VCT plus free 2 year supply of condoms) and informed written consent will be obtained. VCT will also be offered to all respondents regardless of intervention group assignment during the second follow-up period. In accordance with Kenya’s national guidelines for HIV testing, details about HIV (prevention, counseling and testing options and procedures, the disease itself, and treatment possibilities) will be covered in the consent explanations and information leaflet. Individuals 18 years and older will provide their own consent. For individuals between 12 and 18 years of age, parental consent will be sought in addition to the respondents’ consent to testing. No children younger than 12 years will be tested.

Field workers who have been appropriately trained and certified to conduct VCT will perform the pre- and post-test counseling as well as HIV testing at the respondent’s home. A supervisor with extensive VCT experience will be hired for the project. His/her role will be to regularly observe and assess VCT sessions conducted by each Field

Officer, and to ensure the procedure is consistently followed and that respondents receive adequate and appropriate counseling during the sessions.

Field workers will ensure a private location is available before counseling and testing begins. Rooms with a ceiling, a quiet area in the compound or another location away from home may serve as private locations. For those who provide informed consent for the VCT intervention, all three components (pre-test counseling, HIV testing and post- test counseling) will be performed in a single sitting, as is done at national VCT sites. This has been shown to considerably reduce the rate of undelivered HIV results.

The HIV testing procedure will involve serial testing with the rapid HIV tests Determine, Bioline and Unigold as per national guidelines. These tests are readily available and simple to use and interpret. Blood will be collected via finger prick with a sterile lancet, and will first be tested with Determine. Positive results will be confirmed with Bioline. Any respondents with discordant Determine and Bioline test results will have a third rapid, tie-breaker test (Unigold) performed immediately. All 3 tests will be performed using blood from the same finger prick, and thus each respondent will be pricked only once. Respondents who test positive for HIV will be referred to the closest clinic with the capacity to provide HIV/AIDS care and treatment.

*APPLICATION FOR APPROVAL TO USE HUMANS AS EXPERIMENTAL SUBJECTS (STANDARD FORM) – revised 9/25/2007)*

- 5 -

For participants who provide consent for testing but decline to obtain the results, the drops of blood will be placed on filter paper for preparation of a dried blood sample which will then be stored for testing at a later time.

The quality of the VCT sessions will be ensured in the following ways:

1) Proper documentation of consent will be strictly monitored

2) A supervisor will assess counseling sessions of each interviewer at least two half days per month.

3) To ensure quality of the HIV testing, a random sample consisting of 10% of the respondents receiving VCT will be re-tested for HIV. It would be too intrusive to visit a respondent’s home twice within a short period, and to have two different counselors test the respondent twice. Instead, a random sample of 10% percent of participants who receive VCT will also have their drops of blood placed on a filter paper, dried and stored for testing at a later time for quality control purposes.

REVISION TO ABOVE PARAGRAPH (FEBRUARY 2012): DUE TO A RECALL OF THE BIOLINE TESTING INSTRUMENT, THE STUDY WILL USE DETERMINE AND UNIGOLD TO TEST FOR HIV. IN THE EVENT OF DISCORDANCE, ELISA WILL BE USED TO BREAK THE TIE. ELISA TAKES 2 MONTHS TO PRODUCE RESULTS (FROM A LAB), THEREFORE RESEARCH STAFF WILL CONTACT ANY RESPONDENTS REQUIRING THE ELISA TESTS WHEN THE RESULTS COME IN, MEET WITH THEM AND DELIVER THE RESULTS. THIS CHANGE HAS BEEN OUTLINED IN THE ACCOMPANYING AMENDMENT.

e) Anonymous HIV testing

As previously described, VCT will be offered to half of the respondents during the first follow-up period, and to all respondents during the second follow-up period. However, in order to calculate true HIV incidence rates for each of the intervention groups, it would also be necessary to have HIV serostatus data for the respondents assigned to the non- VCT groups during first follow-up. In accordance with internationally accepted ethical standards and UNAIDS/WHO guidelines, we will perform anonymous linked HIV

testing on the blood samples collected during the first follow-up period. The anonymous HIV test will be performed for all respondents who provided informed consent during the first follow-up, using the same blood sample drawn for HSV-2 testing to minimize the number of procedures performed on each respondent.

During the first follow-up period, each blood sample will be labeled with a unique numerical laboratory identifier. The numerical laboratory identifier will be different from the respondent’s study identifier. A master list linking the laboratory identifier to the study identity number (linking data) will be kept in an encrypted file in a password protected computer. The blood sample will be tested for HIV using Vironostika HIV EIA at a government laboratory. Positive tests will be confirmed with a second HIV EIA test (Murex).

*APPLICATION FOR APPROVAL TO USE HUMANS AS EXPERIMENTAL SUBJECTS (STANDARD FORM) – revised 9/25/2007)*

- 6 -

At the end of the study in 2011, when the HIV results are merged with the other sexual, behavioral and demographic data, all personal identifiers will be deleted to preserve complete confidentiality. Thus, the HIV results will be linked only to sexual and behavioral information, but will not be linked to the respondents’ names or geographic locations other than district name. We will therefore not be able to provide the results of this test to the respondents as the sample will not be linked to respondents’ names. However, in accordance with the UNAIDS/WHO guidelines on linked anonymous HIV testing, for those respondents who would like to find out their serostatus, we will provide a referral to the nearest VCT center. In addition, all

respondents will have been offered VCT by the end of the study and thus, all respondents

will have had the opportunity to find out their HIV status if they so choose.

Performing this additional linked anonymous HIV test will provide us with very valuable information on HIV status of most respondents in all intervention groups before and after the intervention (ie at first follow-up and at second follow-up). Thus, we will be able to determine true HIV incidence rates, or the number of new HIV infections that occurred over time, for each of the intervention groups.

f) Anthropometric Measurement and Anemia testing of Women and Children under 5 yrs

Anthropometric data will be collected for children under 5 years of age who were born to female respondents sampled for this study. Weight, recumbent length for infants under 2 years of age, standing height for children above 2 years, and Mid-Upper Arm Circumference (MUAC) will be measured by trained field officers using calibrated

digital scales, stadiometers and infantometers. Mothers’ height, weight and MUAC measurements will also be recorded. Hemoglobin levels for both mother and children under 5 years will be determined using the HemoCue system. The procedure will involve collecting a drop of finger-prick blood (or heel-prick blood in infants) using a sterile lancet, and a microcuvette. The microcuvette containing the drop of blood will be placed in a battery-operated photometer to obtain a rapid measurement of hemoglobin level.

The results of the anemia test will be reported to the respondents, and those with low hemoglobin levels will be informed and advised to seek treatment.

Methodology for Students who are in boarding school or respondents who can't be found at home:

"Camps" will be held in pre-determined locations such as on the grounds of health centers or schools in order to reach subjects who are difficult to reach or unlikely to be found at their homes. Permission to hold the camps at these specific locations will be obtained from relevant parties. Students who are currently attending boarding schools or those respondents who can not be found at home will be invited to attend the camps through letters distributed to parents, teachers and village elders. At the camps, consent will be obtained and surveys will be administered, VCT and condoms will be offered to those sampled and anthropometric measurements will be obtained for female respondents with children under 5 years of age for both the mother and child as per the original protocol. The camps will be organized such that privacy and confidentiality are strictly maintained.

*APPLICATION FOR APPROVAL TO USE HUMANS AS EXPERIMENTAL SUBJECTS (STANDARD FORM) – revised 9/25/2007)*

- 7 -

| All surveys, VCT, condom distribution and anthropometric measurements will take place in a private space such as an empty room within the health facility or school, or in tents that were set up for this purpose. The mobile clinic will also be set up  at the camp so that respondents who consent to the blood draw for HSV-2 and anonymous HIV testing will have their blood drawn prior to leaving the camp.  g) Intense tracking for respondents who could not be found at home or with the camp methodology.  Respondents who could not be found at home or with the camp methodology will be difficult and costly to continue to trace as they may have moved to other areas outside of  our working districts. Due to resource and time constraints, approximately 25% of these  respondents will be sampled for long range, intense tracking. Teams of field officers and  lab technicians will then be sent to various locations (including those outside of our study districts) in order to individually track the sampled respondents at their homes. Once  these respondents are found they will be surveyed and tested for HSV-2 as well as offered anonymous HIV testing. However, due to budgetary constraints, it will not be possible for the respondents found during intense tracking to be followed-up in phase 2. Thus, these respondents will not be part of the sample used to evaluate the VCT and condom interventions. For this reason, we will not conduct the VCT and condom interventions with this particular group of respondents. They will only be surveyed and offered HSV-2 and anonymous HIV testing for the purpose of evaluating the previous school-based HIV prevention programs.  Outcomes  The study will enable us to compare self-reported risky sexual behavior as well as HIV and HSV-2 prevalence in respondents who were randomized to one of 4 school-based HIV educational interventions. We will also be able to assess the impact of VCT and  free condom distribution on self-reported risky sexual behavior as well as HIV and HSV-  2 incidence rates. The third component of the study will evaluate the impact of the Reduced Costs of Education program on the prevalence of anemia, stunting, wasting and underweight in children under 5 years of age born to female respondents. |
| --- |
| **3. Drugs and Devices.** *If the study involves the administration of an investigational drug that is not approved by the Food and Drug Administration (FDA) for the use outlined in the protocol, then the principal*  *investigator (or sponsor) must obtain an Investigational New Drug (IND) number from the FDA. If the study*  *involves the use of an approved drug in an unapproved way the investigator (or sponsor) must submit an application for an IND number. Please attach a copy of the IND approval (new drug), or application (new use.).*  *If the study involves the use of an investigational medical device and COUHES determines the device poses*  *significant risk to human subjects , the investigator (or sponsor) must obtain an Investigational Device and*  *Equipment (IDE) number from the FDA.* |
| **Will drugs or biological agents requiring an IND be used? YES NO**  *If yes, please provide details:*  **Will an investigational medical device be used? YES NO**  *If yes, please provide details:* |
| **4. Radiation** *If the study uses radiation or radioactive materials it may also have to be approved by*  *the Committee on Radiation Exposure to Human Subjects (COREHS). COUHES will determine if you need*  *COREHS approval.* |
| **Will radiation or radioactive materials be used? YES NO**  *If yes, please provide details:* |
| **5. Diets** |
| **Will special diets be used? YES NO**  *If yes, please provide details:* |

**III. HUMAN SUBJECTS**

**1. Subjects**

**A. Estimated number:** 20,000 (approximately)

**B. Age(s): 17-22 years**

*APPLICATION FOR APPROVAL TO USE HUMANS AS EXPERIMENTAL SUBJECTS (STANDARD FORM) – revised 9/25/2007)*

- 8 -

| **C. Inclusion/exclusion criteria**  **i. What are the criteria for inclusion or exclusion?**  **Only students enrolled in grade 6 in 2003 in one of the 328 primary schools involved in the ICS study will be eligible for the Biomarkers follow-up study. ii. Are any inclusion or exclusion criteria based on age, gender, or race/ethnic origin?** *If so, please explain and justify*  Subjects will be enrolled based on their grade and school enrollment in 2003.  This implies this project is mainly targeted toward adolescents, some of whom will be under the age of 18 at the time of the study. |
| --- |
| **D. Please explain the inclusion of any vulnerable population (e.g. children,**  **cognitively impaired persons, non-English speakers, MIT students), and why that population is being studied.**  Since the objective of this study is to measure the impact of various school-based HIV  prevention interventions, it is necessary to conduct data collection activities among adolescents. We will also be collecting anthropometric data, and conducting anemia testing on children under 5 years of age. |
| **2. Subject recruitment** *Identification and recruitment of subjects must be ethically and legally acceptable and free of coercion. Describe below what methods will be used to identify and recruit subjects* |
| Subjects who have been selected for the biomarker follow-up study will be visited at their  current school or at their home or will be invited to attend camps. Field workers will conduct up to 3 visits to meet with sampled respondents.  The purpose and procedure of the follow-up survey (excluding the VCT component and testing for HSV-2 and HIV) will be explained carefully in person to each participant at the time the subject is visited or at the camp. The participants will be given the option to sign a consent form if they agree to participate in the survey, or to refuse to participate. The consent shall be sought under circumstances that provide the prospective subject sufficient opportunity to consider whether or not to participate. Informed consent will also be sought from a parent or legal guardian for all participants who are minors (under the age of 18) at the time of follow-up, unless they are considered to be emancipated (i.e. married and living outside the household). After they have signed the consent form, participants will be reminded that they are free to stop answering the survey questions at any time.  The purpose and procedure of the biomarker tests will be explained carefully in person to each participant at the end of the follow-up survey (or after the participant has refused to participate in the follow-up survey). Consent will be sought separately for VCT, HSV-2, linked anonymous HIV testing, anemia testing and anthropometric measurement. The participants will be given the option to sign a consent form if they choose to participate in a given test, or to refuse to participate. The consent shall be sought under circumstances that provide the prospective subject sufficient opportunity to consider whether or not to participate.  Informed consent will be sought both from the individual and from a parent or legal guardian for participants who are still minors (less than 18 years of age) at the time of the follow-up, unless they are considered to be emancipated (i.e. married and living outside |

*APPLICATION FOR APPROVAL TO USE HUMANS AS EXPERIMENTAL SUBJECTS (STANDARD FORM) – revised 9/25/2007)*

- 9 -

| the household). The informed consent from the parent and guardian will clearly state that  the guardian waives the right to know the result of the test. Parents who do not agree to  this clause will be considered to have refused to participate, and the test will not be conducted on their children. |
| --- |
| **Please attach a copy of any advertisements/ notices and letters to potential subjects** |
| **3. Subject compensation** *Payment must be reasonable in relation to the time and trouble associated with participating in the study. It cannot constitute an undue inducement to participate* |
| **Describe all plans to pay subjects in cash or other form of payment (i.e. gift**  **certificate)**  All subjects in the sample will receive a gift worth approximately $2 when they meet  with the field workers  **Will subjects be reimbursed for travel and expenses?**  Subjects will be reimbursed for travel costs associated with attending the mobile clinics  for HSV-2 testing |
| **4. Potential risks.** *A risk is a potential harm that a reasonable person would consider important in deciding whether to participate in research. Risks can be categorized as physical, psychological,*  *sociological, economic and legal, and include pain, stress, invasion of privacy, embarrassment or exposure*  *of sensitive or confidential data. All potential risks and discomforts must be minimized to the greatest extent possible by using e.g. appropriate monitoring, safety devices and withdrawal of a subject if there is evidence of a specific adverse event.* |
| **What are the risks / discomforts associated with each intervention or procedure in**  **the study?**  The subjects do not risk any legal harm by participating in the research.  Subjects participating in this study may suffer some minor pain associated with the blood collection for the HSV-2, HIV and anemia tests. The blood collection for these tests involves either pricking the fingertip (or heel for young infants) or drawing blood from the cubital vein. Both procedures are very short and involve very little pain or discomfort. Subjects participating in the research will not risk any other physical harm (the procedures followed will ensure sterility of the instruments and use of gloves to  avoid contamination of the respondent or the field officer).  The largest risks to the subjects are psychological harm (upon learning that they are HIV  positive) and social harm if confidentiality is breached, since the information gathered  has the potential to adversely affect the relationship of the respondents with their parents, teachers, partners, and friends.  **What procedures will be in place to prevent / minimize potential risks or discomfort?**  To prevent social harm, strict confidentiality will be ensured (see below).  To prevent psychological harm, HIV testing activities will be conducted by appropriately trained and certified counselors. As described above, the tests will be conducted as part of a Voluntary Counseling and Testing session. Blood for HSV-2 testing will be drawn by experienced nurses or phlebotomists.  Following standard practice, respondents who test positive for HIV will be referred to the closest clinic with the capacity to follow HIV patients. There are several HIV/AIDS programs in the area which distribute Antiretroviral drugs (ARVs) and provide treatment |

*APPLICATION FOR APPROVAL TO USE HUMANS AS EXPERIMENTAL SUBJECTS (STANDARD FORM) – revised 9/25/2007)*

- 10 -

| and care for people infected with HIV (Médecins Sans Frontières in Busia, AMPATH in  Eldoret). We will obtain a guarantee from these organizations that they will accept  participants from our study in their treatment and care programs. Since we have collaborated with these organizations in the past and have an excellent relationship with them, no problems with obtaining this guarantee are foreseen. We will also provide the respondent with a voucher to cover the transportation cost to the program for their first visit. Respondents who test positive for HSV-2 will be referred to the nearest STI clinic for consultation and treatment in accordance with guidelines set by the Kenyan Ministry of Health. |
| --- |
| **5. Potential benefits** |
| **What potential benefits may subjects receive from participating in the study?**  What potential benefits may subjects receive from participating in the study?  Respondents who learn that they are infected with HIV may benefit from the study if the knowledge of their positive status encourages them to seek medical care early and to enroll in an ARV program.  Respondents who learn that they are not infected with HIV may benefit from the study if the knowledge of their negative status, together with counseling, encourages them to further avoid risky behaviors and helps them remain HIV negative.  Respondents who learn that they are infected with HSV-2 will be referred to the nearest STI clinic for consultation and treatment if needed as per the Kenyan Ministry of Health guidelines.  Those women and children who are found to have low hemoglobin levels will also be advised to seek care at the nearest clinic.  **What potential benefits can society expect from the study?**  There is considerable debate as to whether scalable school-based HIV/AIDS education  programs can be effective in limiting the spread of HIV among youth. This study will provide rigorous evidence on the impact of these education programs on trasmission of HIV and STIs. Furthermore, our study will also examine the effectiveness of additional HIV prevention strategies - Voluntary Counseling and Testing for HIV (VCT) and distribution of free condoms. The results of this study will help guide future efforts to combat HIV/AIDS in Kenya and elsewhere. |
| **6. Data collection, storage, and confidentiality** |
| **How will data be collected?**  The data collection will take place during visits by IPA field officers to subjects’ homes, at  mobile clinics or at camps set up by the IPA field workers.  Both the follow-up survey, which will include personal questions about the respondent’s sexual activity, and the VCT session, which will involve a discussion of sensitive topics with the respondent including disclosure of HIV test results to the respondent, will be conducted in a private location, with only the respondent and the counselor present. Field workers will ensure a private location is available before administration of the survey and testing procedures. Rooms with a ceiling, a quiet area in the compound or another location |

*APPLICATION FOR APPROVAL TO USE HUMANS AS EXPERIMENTAL SUBJECTS (STANDARD FORM) – revised 9/25/2007)*

- 11 -

away from home may serve as private locations. In the event that the survey and VCT is administered at a camp, a private location such as a tent or empty nearby room will be used.

For HSV-2 testing, respondents will be invited to attend a mobile clinic set up near their home in a location that ensures privacy for the participants. Mobile clinics will also be held at the camps. This clinic will be run by IPA field workers and a qualified nurse who will be responsible for the blood draws.

Parental consent will be sought for all respondents who are minors at the time of follow-up The parental consent form, however, will also include a clause stating that participant’s survey responses and test results are confidential and will not be disclosed to anyone including the parents of the respondent. No survey and/or tests will be conducted unless the parent has understood and agreed to this clause and has signed the informed consent form.

**Is there audio or videotaping? YES NO *Explain the procedures you plan to follow.***

**Will data be associated with personal identifiers or will it be coded?**

**Personal identifiers Coded *Explain the procedures you plan to follow.***

To preserve confidentiality of the surveys and test results the following steps will be

carried out.

1) To avoid social harm to the respondents in the event that an enumerator’s bag (including the survey and tests results) is stolen or misplaced, no names will appear on the survey or test forms. Instead, numeric study identifiers will be used on any hard copies containing confidential information. A master list linking the names of the individuals to the numeric study identifiers will be kept in an encrypted file in a password protected computer. The field officer will carry copies of survey with the name of the interviewer penciled on the survey, and will erase the name once the interview starts.

2) UNAIDS/WHO guidelines on linked anonymous HIV testing will be closely followed to protect the identity of the participants and prevent disclosure of an individual’s HIV status. For subjects who have chosen to participate in anonymous HIV testing, blood samples taken for HSV-2 testing during the first follow-up period will be assigned and labeled with a unique laboratory number. A master list linking the laboratory number to study identity number (linking data) will be kept in an encrypted file in a password protected computer. When these HIV results are merged with the other data, all personal identifiers will be deleted to preserve complete confidentiality. Thus, the HIV results will never be linked to a respondent’s name or other information which could enable a respondent to be identified.

**Where will the data be stored and how will it be secured?**

Throughout the project, hard copies of the data will be stored in the offices of IPA in Busia

Kenya, where they will be kept during the day and locked at night. The soft copies will be kept in password protected computers, and will not contain individual identifiers.

**What will happen to the data when the study is completed?**

After the completion of the study, hard copies of data will be kept in a locked storeroom

*APPLICATION FOR APPROVAL TO USE HUMANS AS EXPERIMENTAL SUBJECTS (STANDARD FORM) – revised 9/25/2007)*

- 12 -

| owned by IPA in Busia, Kenya. After completion of the data entry and the analysis, the  hard copies will be destroyed. The anonymous soft copies will be passed on to the  principal and associate investigators on the study as well as the research assistants. At a later stage, study data with all individual identifying information (such as name and home location) removed will be incorporated in a data archive with public access.  **Can data acquired in the study affect a subject’s relationship with other individuals (e.g. employee-supervisor, patient –physician, student-teacher, family relationships)?** Some of the data collected from subjects could conceivably affect their relationship with their partners or their parents. To avoid this, field workers will ensure complete privacy during the interview and testing, and will keep all information obtained in the strictest of confidence. Hard copies of the data will be kept in a locked room. |
| --- |
| **7. Deception** *Investigators must not exclude information from a subject that a reasonable person would want to know in deciding whether to participate in a study.* |
| **Will information about the research purpose and design be withheld from subjects?**  **YES NO *If so, explain and justify.*** |
| **8. Adverse effects.** *Serious or unexpected adverse reactions or injuries must be reported to COUHES*  *within 48 hours. Other adverse events should be reported within 10 working days.* |
| **What follow-up efforts will be made to detect any harm to subjects and how will**  **COUHES be kept informed?**  The field workers will spend as much as time as needed in each respondent’s home to  listen to the concerns they may have. COUHES will be informed by the investigators of the study via email or phone if any harm is detected. |
| **9. Informed consent.** *Documented informed consent must be obtained from all participants in studies that involve human subjects. You must use the templates available on the COUHES web-site to prepare*  *these forms. Draft informed consent forms must be returned with this application. Under certain*  *circumstances COUHES may waive the requirement for informed consent.* |
| **Attach informed consent forms with this application.** |
| **10. The HIPAA Privacy Rule.** *If your study involves disclosing identifiable health information about a subject outside of M.I.T., then you must conform to the HIPAA Privacy Rule and complete the questions*  *below. Please refer to the HIPAA section, and to the definitions of protected health information, de-identified data and limited data set on the COUHES web-site.* |
| **Do you plan to use or disclose identifiable health information outside M.I.T.?**  **YES NO**  *If YES, then the subject must complete an Authorization for Release of Protected Health Information Form.*  *Please attach a copy of this draft form. You must use the template available on the COUHES web-site.*  *Alternatively, COUHES may grant a Waiver of Authorization if the disclosure meets criteria outlined on the COUHES web-site.*  **Are you requesting a Waiver of Authorization? YES NO**  *If YES, explain and justify.*  **Will the health information you plan to use or disclose be de-identified? YES NO** |

*APPLICATION FOR APPROVAL TO USE HUMANS AS EXPERIMENTAL SUBJECTS (STANDARD FORM) – revised 9/25/2007)*

- 13 -

**Will you be using or disclosing a limited data set? YES NO**

*If YES, then COUHES will send you a formal data use agreement that you must complete in order for your application to be approved*

**IV. INVESTIGATOR’S ASSURANCE**

**I certify the information provided in this application is complete and correct**

**I understand that I have ultimate responsibility for the conduct of the study, the ethical performance of the project, the protection of the rights and welfare of human subjects, and strict adherence to any stipulations imposed by COUHES**

**I agree to comply with all MIT policies, as well all federal, state and local laws on the protection of human subjects in research, including:**

• **ensuring all study personnel satisfactorily complete human subjects training**

• **performing the study according to the approved protocol**

• **implementing no changes in the approved study without COUHES approval**

• **obtaining informed consent from subjects using only the currently approved consent form**

• **protecting identifiable health information in accord with the HIPAA Privacy**

**Rule**

• **promptly reporting significant or untoward adverse effects**

**Signature of Principal Investigator**

**Date**

**Print Full Name and Title**

**Signature of Department Head**

**Date**

**Print Full Name and Title**

*Please return 3 hard copies of this application (1 with original signatures) to the*

*COUHES office E25-143b.*

*APPLICATION FOR APPROVAL TO USE HUMANS AS EXPERIMENTAL SUBJECTS (STANDARD FORM) – revised 9/25/2007)*

- 14 -
